# A Novel Form of Chondrocyte Stress is Triggered by a COMP Mutation Causing Pseudoachondroplasia

**DOI:** 10.1002/humu.21631

**Published:** 2011-10-17

**Authors:** Farhana Suleman, Benedetta Gualeni, Hannah J Gregson, Matthew P Leighton, Katarzyna A Piróg, Sarah Edwards, Paul Holden, Raymond P Boot-Handford, Michael D Briggs

**Affiliations:** 1 Wellcome Trust Centre for Cell-Matrix Research, Faculty of Life Sciences, The University of ManchesterManchester, United Kingdom; 2 Shriner's Hospitals for Children, Research CenterPortland, Oregon

**Keywords:** pseudoachondroplasia, cartilage oligomeric matrix protein, chondrocyte stress

## Abstract

Pseudoachondroplasia (PSACH) results from mutations in cartilage oligomeric matrix protein (COMP) and the p.D469del mutation within the type III repeats of COMP accounts for approximately 30% of PSACH. To determine disease mechanisms of PSACH in vivo, we introduced the *Comp* D469del mutation into the mouse genome. Mutant animals were normal at birth but grew slower than their wild-type littermates and developed short-limb dwarfism. In the growth plates of mutant mice chondrocyte columns were reduced in number and poorly organized, while mutant COMP was retained within the endoplasmic reticulum (ER) of cells. Chondrocyte proliferation was reduced and apoptosis was both increased and spatially dysregulated. Previous studies on *COMP* mutations have shown mutant COMP is co-localized with chaperone proteins, and we have reported an unfolded protein response (UPR) in mouse models of PSACH-MED (multiple epiphyseal dysplasia) harboring mutations in *Comp* (T585M) and *Matn3, Comp etc* (V194D). However, we found no evidence of UPR in this mouse model of PSACH. In contrast, microarray analysis identified expression changes in groups of genes implicated in oxidative stress, cell cycle regulation, and apoptosis, which is consistent with the chondrocyte pathology. Overall, these data suggest that a novel form of chondrocyte stress triggered by the expression of mutant COMP is central to the pathogenesis of PSACH. Hum Mutat 33:218–231, 2012. © 2011 Wiley Periodicals, Inc.

## Introduction

Pseudoachondroplasia (PSACH; MIM# 177170) is an autosomal dominant disease that affects skeletal development and has a clinical spectrum ranging from mild to severe [Maroteaux et al., 1980; Rimoin et al., 1994; Wynne-Davies et al., 1986]. The disease results almost exclusively from mutations in the gene encoding cartilage oligomeric matrix protein (COMP; MIM# 600310) [Briggs and Chapman, 2002; Briggs et al., 1995; Hecht et al., 1995], a 550-kDa extracellular matrix (ECM) glycoprotein found in cartilage, tendon, and ligament [DiCesare et al., 1994; Hecht et al., 1998; Oldberg et al., 1992]. Mutations in *COMP* can also result in a related chondrodysplasia, multiple epiphyseal dysplasia (MED; MIM# 132400), which is a clinically variable and genetically heterogeneous disease that can also result from mutations in the genes encoding matrilin-3 (*MATN3*; MIM# 602109) and type IX collagen (*COL9A1*; MIM# 120210, *COL9A2*; MIM# 120260, and *COL9A3*; MIM# 120270) [Briggs and Chapman, 2002; Chapman et al., 2001; Muragaki et al., 1996; Paassilta et al., 1999].

COMP is thought to be important for ECM assembly within the cartilage growth plate by acting as a catalyst in the regulation of collagen fibril assembly [Halasz et al., 2007; Rosenberg et al., 1998], as well as interacting with other ECM proteins such as members of the matrilin protein family [Fresquet et al., 2007; Mann et al., 2004] and type IX collagen [Holden et al., 2001; Thur et al., 2001]. COMP has also been shown to be central to the integrity of chondrocytes within the ECM. In the first instance, it is important in the regulation of chondrocyte attachment to the ECM through integrin receptors [Chen et al., 2005; Rock et al., 2010]. Second, COMP plays an important regulatory role in chondrocyte proliferation [Xu et al., 2007] and has recently been proposed to have a cell survival role by promoting the upregulation of inhibitors of apoptosis proteins (IAPs), particularly XIAP, cIAP1, cIAP2, and survivin [Gagarina et al., 2008]. Overall, these studies have suggested an important function for COMP in the regulation and maintenance of cell–matrix integrity within the cartilage growth plate.

It was therefore surprising that a knockout mouse model of COMP displayed no overt skeletal phenotype [Svensson et al., 2002], which supports the hypothesis that the compromised skeletal development in PSACH and MED results from antimorphic (dominant negative) *COMP* mutations [Briggs and Chapman, 2002].

COMP is a modular protein and comprises an N-terminal oligomerization domain, four epidermal growth factor-like (TSP2) repeats, eight calcium binding type 3 (TSP3) repeats, and a C-terminal globular domain. An increasing number of mutations (∼15%) have been reported in the C-terminal domain of COMP [Kennedy et al., 2005b]; however, the majority of PSACH and MED mutations (85%) are clustered in the TSP3 repeats [Kennedy et al., 2005a]. The most common disease-causing mutation is the in-frame deletion of an aspartic acid residue (p.D469del) from the seventh T3 repeat (T3_7_), which accounts for approximately 30% of all PSACH [Briggs and Chapman, 2002; Hecht et al., 1995; Ikegawa et al., 1998]. This archetypal mutation has therefore been extensively studied both in vivo using patient cartilage and in vitro using cell culture models; reviewed in Posey et al. [2008]. These studies have consistently shown that p.D469del (and other T3 mutations) result in the retention of mutant COMP protein within the rER of chondrocytes, along with matrilin-3 and collagen type IX, while a number of chaperone proteins are co-localized with the retained mutant COMP [Hecht et al., 2005, 2001]. In addition, cartilage from PSACH patients has a disorganized ECM and there is an increase in cell death in vivo [Hecht et al., 2004], which can be recapitulated in vitro by cell culture models [Duke et al., 2003; Hashimoto et al., 2003].

While cell culture models have recapitulated some of the pathological features in vitro, they are not adequate models to dissect the disease pathways involved in PSACH. Recently, two mouse models of D469del COMP have been described that display some of the pathological features of PSACH [Posey et al., 2009; Schmitz et al., 2008]. These are transgenic models in which mutant COMP is overexpressed and may therefore not be the most physiologically relevant model to dissect the disease mechanisms of PSACH in vivo. In order to study the disease pathology of PSACH in a more physiologically relevant model, we report the generation and phenotypic description of the first knock-in mouse model generated by homologous recombination and harboring the common D469del COMP mutation. This model closely recapitulates the human disease pathology and provides a phenotypically and pathologically relevant model in which to study the disease pathways that are involved in PSACH.

Here, we provide the first report of the cellular consequences of the expression of D469del COMP in vivo and demonstrate that reduced cell proliferation and increased chondrocyte apoptosis is associated with a downregulation of peroxiredoxin 2, a gene important for protecting against oxidative damage and in regulating apoptosis.

## Materials and Methods

### Generation of *Comp* D469del Knockin Mice

A 129S6/SvEvTac mouse spleen genomic DNA library was previously screened for a clone containing the entire *Comp* gene [Pirog-Garcia et al., 2007]. A *loxP*-flanked *Neo* cassette was cloned into pBluescript (pBS-Neo) using an *Eco*RV site in the polylinker sequence. The long arm of the construct was generated by *Eco*RI digestion and subcloning of an 8-kb genomic fragment containing *Comp*. The short arm of the construct (2 kb) was generated by subcloning of an *Eco*RI/*Spe*I *Comp* positive fragment (6 kb). The *Comp* D469del change (TGACGATGAT—AATG) was introduced by site-directed mutagenesis of a 1.6-kb fragment generated by restriction digestion using a *Spe*I site in the pBluescript polylinker and a *Mlu*I site in the construct. The primers used for the introduction of the mutation were; forward (mutation): 5′-T GAC GAT GAT—AAT GAC GGA G-3′, reverse (mutation): 5′-C CCT GTC TTA—ATC ATC GTC A -3′, forward: 5′-GAT GCC TGC GAC AAC TGC C-5′, and reverse: 5′- CCT TGT CAG CAT CGA AC-3′. The presence of the mutation was confirmed by DNA sequencing. In the short arm of the construct, an *Eco*RI restriction site was modified into a *Cla*I site by ligation of a linker oligonucleotide (5′-GAATTATCGATAATTC-3′). The pBluescript vector was modified by *Not*I digestion and Klenow blunting, thus generating a unique *Fse*I site for construct linearization.

R1 (129Sv) embryonic stem (ES) cells were grown on a layer of G418 (Invitrogen) resistant embryonic feeder cells in a medium supplemented with leukemia inhibitory factor. The targeting vector (70 µg) was linearized with *Fse*I and electroporated into 4 × 10 R1 (129Sv) ES cells using a BioRad gene pulser (0.8 kV, 3 µF, 0.1 ms; Bio-Rad Laboratories Ltd). Electroporated cells were propagated in selective medium with G418 (500 µg/ml) for 5–6 days and resistant clones were picked and screened for homologous recombination by *Hin*dIII digestion and Southern blot analysis with an external 1-kb probe. A probe directed against the *Neo* cassette was used to confirm single incorporation of the construct sequence in the mouse genome. The presence of the mutation was confirmed by direct sequencing and the positive clones were grown from frozen stocks and microinjected into C57BL/6 blastocysts and implanted into foster mothers. The resulting chimeric animals were assessed on fur color and mated with a deleter *Cre* line of transgenic mice to delete the *loxP*-flanked selection cassette. Heterozygous F1 offspring were then mated to generate the animals used in all of the experiments described. *Cre* recombination resulted in one *loxP* site remaining in the intronic sequence, allowing for genotyping from tail-tip genomic DNA using primers spanning intron 16 (Extract-N-AmpTM Tissue PCR Kit, Sigma–Aldrich Ltd., Haverhill, UK).

### Generation of the COMP D469del Cell Line

Wild-type and mutant (p.D469del) full-length human COMP cDNA (nucleotides 1–2,271 and including the native signal peptide but missing the stop codon; GenBank accession number NM_000095.2 with nucleotide 1 counted as the first nucleotide of the translation initiation codon) was cloned in-frame into the pEGFP-N3 vector (Takara Bio/Clontech, Saint-Germain-en-LayeFrance) to engineer a green flourescent protein (GTF) tag at the C-terminus. T25 flasks of 75% confluent HT1080 cells were transfected with 2 µg of vector DNA using Lipofectin and recombinant cells selected using G418. Following selection stably transfected cells were fluorescence-activated cell sorting (FACS) sorted to enrich for those cells expressing high levels of the transgene.

### Skeletal Preparations and Bone and Growth Rate Measurements

Individual bone lengths and hip angles were measured from radiographic images taken of 3-, 6-, and 9-week-old mice using propriety software (Certus Technology Associates Limited, Exeter, UK). Mice were weighed at 3, 6, and 9 weeks of age and these measurements were used to generate growth curves. All the measurements were analyzed by one-way ANOVA for statistical significance.

### Tissue Extraction and Processing for Histology and Immunohistochemistry (IHC)

Whole femur and tibia knee joints were dissected and fixed for 24 hr in 95% ethanol/5% acetic acid. Tissue samples were then decalcified for up to 2 weeks in 20% EDTA, pH 7.4. Samples were processed overnight in a tissue processor (Microm/Thermo Fisher Scientific Inc., Walldorf, Germany) and embedded in paraffin wax. Six micrometer Sagittal sections were cut on a microtome (Microm/Thermo Fisher Scientific Inc.) and collected on positive slides (superfrost; VWR International Ltd., Lutterworth, UK). Tissue sections were then used for histochemical and immunohistochemical staining. Slides were dewaxed in xylene, rehydrated in a graded alcohol series, and H&E stained using an automated stainer (ThermoShandon Ltd., Runcorn, UK) or used for IHC as described previously [Leighton et al., 2007].

### Ultrastructural Analysis Using Electron Microscopy

Tibias from 7-day-old mice were fixed overnight and processed as previously described [Pirog-Garcia et al., 2007]. Seventy nanometer sections were cut and stained with 0.3% (w/v) lead citrate and images were collected on a FEI Tecnai 12 Biotwin electron microscope (FEI UK Limited, Cambridge, UK).

### Cell Proliferation and Apoptosis Assays

Mice were injected intraperitoneally with BrdU labeling solution (GE Healthcare Life Sciences, Amersham, UK) and sacrificed after 2 hr. Knee joints were dissected and processed as previously described [Pirog-Garcia et al., 2007]. IHC was performed using an anti-BrdU antibody (1:100; Abcam, Cambridge, UK) with a biotinylated secondary rabbit anti-rat IgG, (1:200; Dako cytomation, Cambridge, UK). Images of the growth plate were collected using an Axiovision microscope software (Carl Zeiss Ltd., Welwyn Garden City, UK) and the proportion of BrdU positive cells (brown stain) compared to the total number of cells (Dapi stain) in the proliferative zone was determined for each genotype. The results were quantified using one-way ANOVA.

Apoptosis was visualized using the DeadEnd™ fluorometric TUNEL assay (Promega UK, Southampton, UK) with citrate buffer used for unmasking [Pirog-Garcia et al., 2007]. Images were collected using an Axiovision microscope software (Carl Zeiss) and statistic analysis performed by independent sample *t*-test.

### Real Time qRT-PCR

Cartilage was dissected from 5-day-old mice (rib) or 3-week-old mice (xiphoid) of pooled mixed gender litters and snap frozen in liquid nitrogen. The tissue samples were then homogenized in TRIzol (Invitrogen, UK) using a micro-dismembranator (2,000 rpm for 2 min) and the RNA was isolated as per the manufacturer's protocol. RNA concentration was determined by a Nanodrop ultra-low-volume spectrophotometer. RNA samples of 0.5 µg were subsequently used to synthesize first strand cDNA using superscript III (Invitrogen) and real-time analysis of wild-type and mutant mRNA levels was performed using a SYBR® Green Kit on a ABIPrismTM 7000 sequence detector system (Applied Biosystems, Warrington, UK) as described previously [Leighton et al., 2007; Nundlall et al., 2010; Pirog-Garcia et al., 2007]. Primer sequences were: Grp78/BiP: 5′-GGCACCTTCG-ATGTGTCTCTTC-3′ and rev: 5′-TCCATGACCCGCTGATCAA-3′; Grp94: 5′-TAAGCTGTATGTACGCCGCGT-3′ and rev: 5′-GGAGATCATCGGAATCCACAAC-3′; Cnx: 5′-TGA TTT CCT CTC CCT CCC CTT-3′ and rev: 5′-CAC TGG AAC CTG TTG ATG GTG A-3′; Crt: 5′-GCT ACG TGA AGC TGT TTC CGA-3′ and rev: 5′-ACA TGA ACC TTC TTG GTG CCA G-3′; Erp72: 5′-AGT ATG AGC CCA GGT TCC ACG T-3′ and rev: 5′-AGA AGT CTT ACG ATG GCC CAC C-3′. Each experiment included “no template” controls, was run in duplicate, and had an 18S RNA control. Each experiment was repeated three times for statistical relevance and the results analyzed by independent samples *t*-test.

### SDS-PAGE and Western Blotting

Rib cartilage from 5-day-old mice or xiphoid cartilage from 3-week-old mice was homogenized in 100 µl dH_2_O per 5 mg tissue, boiled in SDS loading buffer containing dithiothreito (DTT) and 40 µl of total protein was loaded on an SDS-PAGE gel. The gel was electroblotted onto a nitrocellulose membrane, which was blocked overnight with 2% skimmed milk powder in phosphate buffered saline-triton (PBS-T). The COMP antibody (GeneTex Inc/Patricell Ltd., Nottingham, UK) was used at a dilution of 1:100 and antibodies to chaperones associated with the UPR were used at a dilution of 1:500 (Grp78, Grp94, Erp72; all from Santa Cruz Biotechnology, Inc., Santa Cruz, CA, USA). The Western blots were scanned and analyzed by densitometry using the AIDA32 software and then normalized to Ponceau staining for total protein (*n* ≥ 3 litters per genotype [∼5–8 mice per litter of mixed gender] and ≥3 separate experiments were performed in duplicate).

Adherent HT1080 cells transfected with GFP vector alone, GFP-COMP wild-type or GFP-COMP mutant were cultured in T75 flasks in DMEM4 (Sigma-Aldrich Ltd., Haverhill, UK) supplemented with 10% FBS (Fisher Scientific UK Ltd., Loughborough, UK), 1% Penicillin/Streptomycin 100× solution (Sigma-Aldrich), 1% of 200 mM L-Glutamine solution (Sigma-Aldrich), 1% nonessential amino acids 100× solution (Sigma-Aldrich), and 360 mg/L G418, changing medium every second day. After they reached confluence, cells were cultured in the same medium for 48 or 72 hr, washed three times in ice-cold PBS and lysed in 1 ml of RIPA buffer (150 mM NaCl, 1% Na Deoxycholate, 0.1% SDS, and 10 mM Tris-HCl, pH 7.4) on ice for 15 min. Cell lysates were centrifuged at 13,000 *g* for 10 min at 4°C and the supernatants were collected. An aliquot of the supernatant was used for protein concentration evaluation. Cell lysates were then boiled for 5 min in SDS-PAGE loading buffer containing DTT. Twenty micrograms of proteins were loaded on an SDS-PAGE 4–12% precast gel (Invitrogen) and electroblotted on a nitrocellulose membrane, which was blocked overnight with 2% skimmed milk powder in PBS-T. The monoclonal COMP antibody (clone 12C4) was used at a dilution of 1:1000; the Polyclonal PRDX2 antibody (ProteinTech Europe, Manchester, UK) and the monoclonal β actin antibody (Abcam, Cambridge, UK) were used at a dilution of 1:500.

### Microarray Hybridization and Statistical Analysis

For the microarray analysis, RNA was isolated using TRIzol (Invitrogen, UK) from the rib chondrocytes of newborn and 5-day-old mice [Nundlall et al., 2010], and the cell culture model at 48 hr after confluence. In order to analyze gene expression in mice that were homozygous for *Comp* D469del, hybridizations were performed on three separate RNA samples (3 × mutant and 3 × wild-type) isolated from chondrocytes pooled from five littermates of mixed gender. Only one RNA sample (one × mutant litter and one × wild-type litter of mixed gender) was examined from mice heterozygous for *Comp* D469del or homozygous for *Matn3* V194D [Nundlall et al., 2010].

RNA quality was assessed using the RNA 6000 Nano Assay and analyzed on an Agilent 2100 Bioanalyser (Agilent Technologies UK Ltd., Edinburgh, UK). RNA concentration was quantified using a Nanodrop ultra-low-volume spectrophotometer (Nanodrop Technologies, Wilmington, DE, USA). The hybridization cocktail was hybridized to either the Mouse 430_2 oligonucleotide array or Human HG-U133_plus_2 array (Affymetrix UK Ltd., High Wycombe, UK) according to the manufacturers' instructions. Arrays were read using an Agilent GeneArray scanner 3000 7G and processed using Affymetrix GCOS (V1.4) software. Statistical analysis was performed using Robust Multi-array Average (RMA), which is an algorithm used to generate expression profiles from Affymetrix data [Irizarry et al., 2003].

## Results

### Generation of the Comp D469del Knockin Mouse Line

Mice harboring the *Comp* D469del mutation in the TSP3 region of COMP were generated by homologous recombination in R1 ES cells using an appropriate targeting strategy (Supp. Fig. S1). The “long arm” of the targeting construct (8 kb) contained exons 1–16 of *Comp* and the “short arm” of the construct (2 kb) contained exons 17–19 of *Comp* (Supp. Fig. S1A). The 3-bp deletion required to produce the D469del mutation was introduced into exon 13 of *Comp* by site-directed mutagenesis. Three hundred and sixty ES cell clones that had been electroporated with the targeting construct and selected by antibiotic resistance were analyzed for homologous recombination using Southern blotting with an external probe (Supp. Fig. S1B). The presence of the mutation was confirmed by direct sequencing of DNA from ES clones that had successfully undergone homologous recombination. Positive clones resulting from the targeting procedure were microinjected into C57BL/6 blastocysts and then implanted into pseudopregnant foster mothers. The resultant chimeric mice were used for the foundation of a transgenic line and a breeding strategy was used to obtain wild-type mice and mice that were either heterozygous or homozygous for the mutation. The presence of the mutation was again confirmed in mice that were homozygous for D469del (Supp. Fig. S1C).

### COMP mRNA and Protein Expression Levels are Comparable between Wild-Type and Mutant Mice

Cartilage from 3-week-old mice was used for RNA extraction and for SDS-PAGE, and Western blot analysis of total protein to confirm that the D469del mutation did not affect the expression of either *Comp* alleles. Prior to qRT-PCR, first strand cDNA was analyzed to ensure that there was no contamination from genomic DNA. This was achieved by performing PCR using a primer pair spanning intron 15 of the COMP gene, which confirmed that there was no contamination from genomic DNA (Supp. Fig. S1D). Thereafter, qRT-PCR using *Comp-*specific primers was performed and this analysis confirmed comparable *Comp* mRNA levels in chondrocytes from wild-type and mutant mice (0.92 ± 0.09; wt vs. mut). Total COMP protein levels in cartilage were determined by SDS-PAGE analysis of DTT-reduced total protein samples followed by Western blotting using a COMP-specific antibody. Protein bands corresponding to wild-type and mutant COMP were present at approximately 100 kDa, which corresponded to the COMP monomer (Supp. Fig. S1E). Wild-type and mutant cartilage protein samples showed comparable levels of COMP when normalized to Ponceau staining and quantified by densitometry (data not shown). Furthermore, protein samples run under non-reducing conditions demonstrated that mutant COMP formed the correct pentameric structure (data not shown).

### Mutant Mice are Normal at Birth but Develop a Progressive Short-Limb Dwarfism and Hip Dysplasia

A normal Mendelian distribution of 1:2:1 (wt/wt:m/wt:m/m) was observed in litters produced from crosses of mice heterozygous for the *Comp* D469del mutation. There were no overt skeletal defects observed at birth in mice of any genotype (data not shown), an observation that is consistent with human PSACH patients when the clinical presentation only becomes apparent in early childhood.

To quantify changes in growth that were caused by the *Comp* D469del mutation, littermates of all three genotypes were weighed at 3, 6, and 9 weeks of age and these measurements were used to prepare growth curves (Supp. Fig. S2). At 3 weeks of age, the body weights of all the mice were comparable, but by 6 weeks of age the body weights of male mice homozygous for the *Comp* D469del mutation were >7% (*P* < 0.05) less that their wild-type and heterozygous littermates. By 9 weeks of age, the differences in weight were statistically significant for both male and female mice homozygous for the mutation (males ∼10%, *P* < 0.02 and females ∼8%, *P* < 0.05).

**Figure 1:**
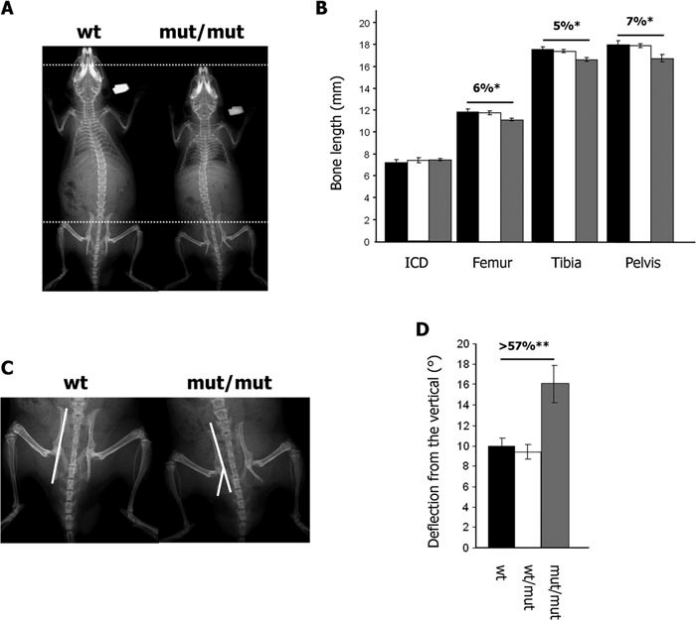
Mutant COMP mice are normal at birth but develop short-limb dwarfism by 9 weeks of age. **A:** Individual mice were radiographed at 3, 6, and 9 weeks of age; radiographs of 9-week-old mice are shown. **B:** Bone measurements of male littermates at 9 weeks of age with inner canthal distance (ICD) used as a marker of intramembranous ossification, and the femur, tibia, and pelvis lengths used as markers of endochondral ossification. The differences in femur, tibia, and pelvis lengths between wild-type males and males homozygous for the mutation were >6%, >5%, and >7%, respectively (*n* ≥ 7 mice per genotype; One-way ANOVA, *P < 0.05). **C:** The angle (°) between the tuberosity of the ischium and the pelvic region was measured at 3, 6, and 9 weeks of age in mice of all three genotypes. **D:** The hip angle was >57% greater for males homozygous for the mutation compared to their wild-type littermates at 9 weeks (*n* ≥ 7 mice per genotype; One-way ANOVA, **P < 0.005). wt, wild-type; wt/m, mice heterozygous for the mutation; m/m, mice homozygous for the mutation.

Radiographs of individual mice of all three genotypes were collected at 3, 6, and 9 weeks of age and bone length measurements were performed (Fig. 1 and Supp. Fig. S3). To determine the effect of the mutation on intramembraneous ossification, skull length and inner canthal distance (ICD) were measured; while the lengths of the pelvis, femur, and tibia were used to determine the effect of the mutation on endochondral ossification. At 9 weeks of age, the mice homozygous for the mutation appeared shorter than their wild-type littermates and the final reduction in adult male bone lengths, relative to wild-type controls, was 6% (*P* < 0.05) for the femur, 7% (*P* < 0.05) for the pelvis, and 5% (*P* < 0.05) for the tibia (Fig. 1B). In female mice, the final reduction in adult bone lengths was 6% (*P* < 0.05) for the femur, 3% (*P* < 0.001) for the pelvis, and 6% (*P* < 0.05) for the tibia (data not shown). In contrast, there were no differences in either the skull length or ICD for mice of all three genotypes and at all time points analyzed (Fig. 1B and data not shown).

Patients with PSACH develop a waddling gait during childhood and we were interested to determine if *Comp* D469del mutant mice developed a hip dysplasia similar to that reported for *Comp* T585M mutant mice [Pirog-Garcia et al., 2007]. In order to determine the effect of the mutation on the development of the pelvis, the angle of deflection from the vertical of the tuberosity of the ischium was measured at 3, 6, and 9 weeks of age in mice of all three genotypes (Fig. 1C; only 9 weeks is shown). Interestingly, at 9 weeks of age the angle at the hip was greater in mice homozygous for the mutation when compared to their wild-type or heterozygous littermates. In males, the angle of deflection was increased by >57% (*P* < 0.01) (Fig. 1D; 10° in wild-type mice compared to 16° in mice homozygous for the mutation). In female mice, there was a >48% (*P* < 0.05) increase in the angle of deflection from 10.4° in wild-type mice to 15.4° in mice homozygous for the mutation (data not shown).

### Mutant Mice Exhibit Abnormal Growth Plate Morphology Characterized by Disorganized Chondrocyte Columns and Areas of Hypocellularity in the Proliferative Zone

**Figure 2:**
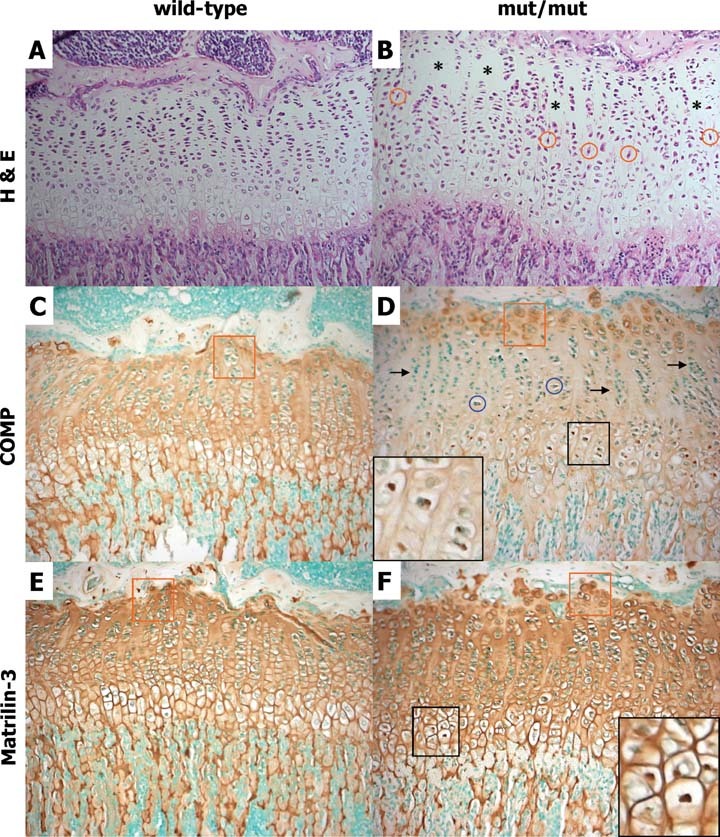
The organization of the growth plate in mutant mice is disrupted by 3 weeks of age and shows marked hypocellularity along with the mislocalization of cartilage structural proteins. Representative images from the growth plates of 3-week-old wild-type and mutant mice. **A and B:** H&E staining showed disruption to the growth plate by 3 weeks of age in mice homozygous for the *Comp* D469del mutation. This disruption was characterized by fewer and more disorganized columns of chondrocytes in the proliferating zone, with distinct areas of hypocellularity (*) and individual chondrocytes that were at right angles to their usual horizontal plane (red circles). IHC using COMP (**C and D**), and matrilin-3 (**E and F**) antibodies revealed less staining in the ECM between the proliferating columns in the growth plates of mice carrying the D469del mutation. Furthermore, there was intracellular staining for mutant COMP and matrilin-3 in chondrocytes from mice homozygous for D469del (insert) and chondrocytes in the mutant growth plates had lost the “clear” appearance of their pericellular matrix (black arrows). Extracellular staining for COMP and matrilin-3 in the resting zone of the mutant growth plates was restricted to the pericellular matrix surrounding chondrocytes (**D and F**; red boxes) compared to the wild-type growth plates (**C and E**; red boxes). mut/mut, mice homozygous for D469del.

By 3 weeks of age, the growth plates of wild-type mice were well organized with ordered columns of proliferating chondrocytes, while a distinct hypertrophic zone was visible that was terminated by a regular vascular invasion front (Fig. 2A). In contrast, mice homozygous for the mutation had an abnormal growth plate characterized by disorganized chondrocytes within individual chondrons, while the chondrons themselves were also misaligned within each column and in some cases individual chondrocytes were seen to be aligned at right angles to their usual horizontal plane (Fig. 2B; red circles). Most striking however were the obvious areas of hypocellularity in the resting and upper proliferative zones of the mutant growth plates (Fig. 2B; asterisks). Furthermore, there appeared to be no clear distinction between the proliferative and hypertrophic zones of the growth plate and the vascular invasion front was also irregular. The growth plates of mice heterozygous for the mutation were comparable to those of wild-type mice in that they had an organized growth plate with clear proliferative and hypertrophic zones and a regular vascular invasion front, however, some regions of hypocellularity were observed within the proliferative zone indicating an intermediate phenotype (data not shown).

### Mutant COMP is Retained within Growth Plate Chondrocytes Along with Matrilin-3 and to a Lesser Extent Type IX Collagen

IHC was performed on the tibia growth plates of 3-week-old mice to determine the localization of key structural components, in particular COMP, matrilin-3 and type IX collagen, which are known to interact with each other. Extracellular staining for COMP was observed in the growth plates of wild-type mice (Fig. 2C) and mice heterozygous for *Comp* D469del (not shown). However, extracellular staining of COMP was markedly reduced in the growth plates of mice homozygous for the mutation, in particular within the proliferative zone, where chondrocytes were also seen to have lost the “white” appearance of their pericellular matrix (Fig. 2D; arrows). In contrast, chondrocytes within the resting zone of mutant growth plates appeared to have more intense staining for COMP in the pericellular matrix compared to wild-type (Fig. 2C and D; red squares). Most notable, however, was the intracellular staining for mutant COMP, which was most pronounced in chondrocytes within the hypertrophic zone (Fig. 2D; insert), although a few proliferative chondrocytes also had retained mutant COMP (Fig. 2D; blue circles).

Extracellular staining for matrilin-3, which was comparable to COMP staining, was observed in wild-type mice and in mice heterozygous for the mutation (Fig. 2E, not shown). Although ECM staining for matrilin-3 was present in the growth plates of mice homozygous for the *Comp* D469del mutation, there was also some intracellular staining seen in the hypertrophic chondrocytes, although this staining was less intense than that seen for mutant COMP (Fig. 2E; insert). Interestingly, there was a similar concentration of matrilin-3 staining in the pericellular matrix surrounding chondrocytes in the resting zone of the mutant growth plate that we had observed with mutant COMP (Fig. 2E and F; red boxes).

Finally, a small amount of intracellular staining was also observed for type IX collagen in chondrocytes from the growth plates of mice homozygous for the mutation when compared to those of heterozygous mice and wild-type controls (Supp. Fig. S4A and B). Furthermore, in mice of all three genotypes staining for type IX collagen was more pronounced in the resting and early proliferative zones, but there were no differences in the localization of type IX collagen in the ECM of mutant mice as seen with both COMP and matrilin-3.

### The Growth Plates of Mutant Mice Also Have Mislocalization of Type II and Type X Collagen

Type II and type X collagen are essential for correct skeletal development and are important cartilage markers; in particular type X collagen, which is expressed exclusively by hypertrophic chondrocytes [Morrison et al., 1996]. Wild-type and mutant growth plates showed no overt differences in the extracellular staining for type II collagen (Supp. Fig. S4C and D) and as expected type X collagen staining was observed only in the ECM of the hypertrophic zone for mice of all three genotypes, however, staining in the growth plates of mice homozygous for the mutation was uneven and appeared diffuse, particularly at the proliferative–hypertrophic boundary (Supp. Fig. S4E and F).

### Ultrastructural Analysis Confirms An Enlarged rER in Mutant Chondrocytes and Abnormal Changes to ECM Morphology in Mutant Cartilage

**Figure 3:**
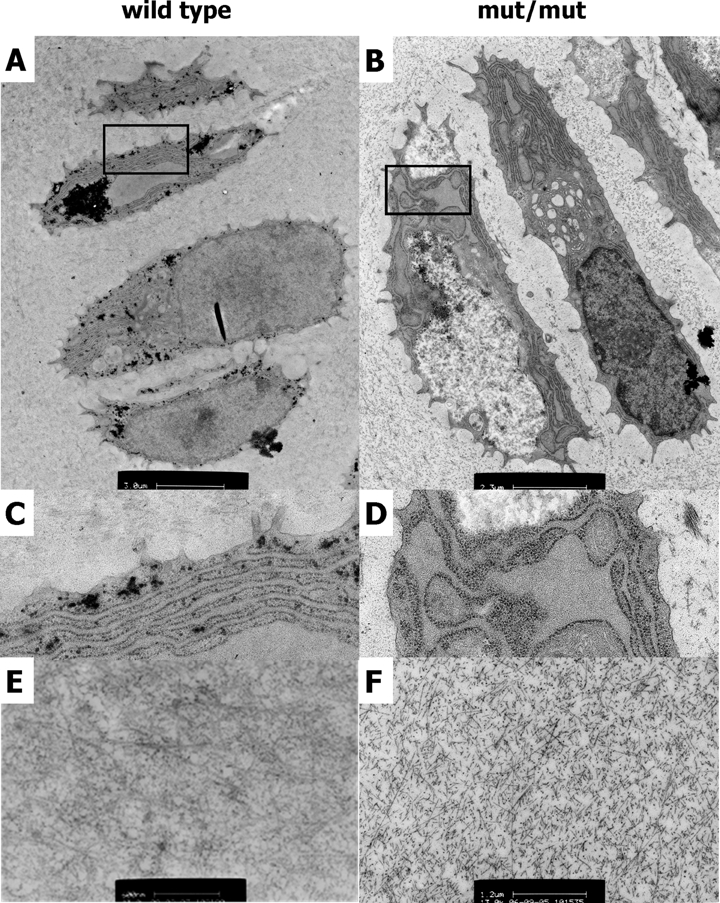
Chondrocytes have dilated cisternae of ER and the ECM ultrastructure is altered in mutant growth plates. **A and B**: These representative images were taken from a complete TEM montage of a day 7 tibia growth plate (from resting to mineralization zones) and show four cell chondrons from the proliferative zone. Chondrocytes from mutant mice show enlarged individual cisternae of ER (**D**) compared to the wild-type mice in which the ER has a typical ribbon appearance (**C**). **E and F**: The ultrastructure of the interterritorial matrix at 1 week of age is altered in mut/mut growth plates and is characterized by more prominent appearing collagen fibrillar material. Scale bars are 3.0 µm (**A**), 2.3 µm (**B**), or 1.2 µm (**E and F**). mut/mut, mice homozygous for D469del.

The tibia growth plates from 7-day-old mice were studied by transmission electron microscopy (TEM) to observe individual chondrocytes and their associated ECM (Fig. 3). While the ER of wild-type chondrocytes appeared within normal limits (Fig. 3A and C), most chondrocytes in the growth plates of mice homozygous for the *Comp* D469del mutation showed enlarged cisternae of ER that was consistent with the retention of mutant COMP and other proteins (Fig. 3B and D). Hypertrophic chondrocytes from wild-type mice were rectangular in shape and the condensation of chromatin was clearly visible. In contrast, hypertrophic chondrocytes from mice homozygous for the mutation were generally irregular in shape and showed the persistence of protein within remnants of the ER (data not shown). In addition, there were clear changes in the morphology of the ECM in the pericellular region of the cartilage growth plate (Fig. 3E and F). This included more pronounced appearing collagen fibrils, which is consistent with a reduction in the levels of fibril surface-associated material (such as COMP) as seen in the other mouse models of PSACH-MED [Leighton et al., 2007; Pirog-Garcia et al., 2007].

### Chondrocyte Proliferation Is Decreased in Mutant Mice While Apoptosis Is Increased and Spatially Dysregulated

The spatial and temporal regulation of chondrocyte proliferation and differentiation within the growth plate is crucial for correct bone growth. Therefore, to help identify a mechanistic link between the expression of *Comp* D469del and reduced longitudinal bone growth, we studied the relative rates of chondrocyte proliferation and apoptosis in the growth plate.

**Figure 4:**
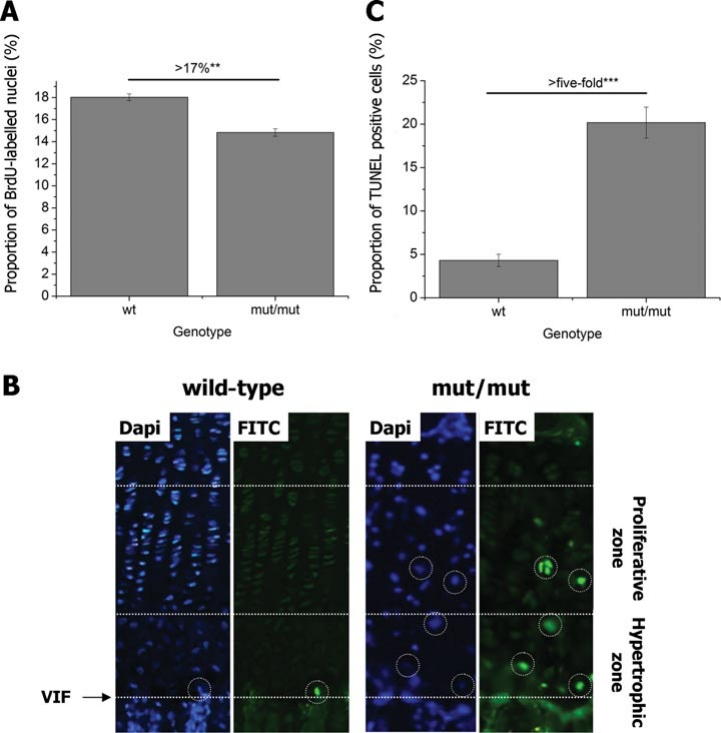
Cell proliferation is decreased and apoptosis is both increased and spatially dysregulated in 3-week-old tibias of mutant mice. **A:** Chondrocyte proliferation, assayed by 2 hr BrdU labeling, was significantly reduced in growth plates of mice homozygous for the D469del mutation (by ∼17%; *n* = 36 sections from three mice per genotype, independent samples *t*-test, **P < 0.005). **B:** TUNEL positive (apoptotic) cells (white circles) were detected in the proliferative and hypertrophic zones of growth plates from mice homozygous for the D469del COMP mutation, but not in their wild-type littermates. The white lines mark the boundaries between the vascular invasion front, hypertrophic, and proliferative zones in the growth plate, and DAPI staining was used as a nuclear counterstain. **C:** Quantification of the TUNEL assay results showed a significant increase in apoptosis in the hypertrophic (>fivefold) zone of growth plates from mice homozygous for the mutation (*n* = 36 sections from three mice per genotype, independent samples *t*-test, ^***^P < 0.001). wt, wild-type; mut/mut, homozygous for D469del; VIF, vascular invasion front.

To determine the effect of mutant *Comp* expression on chondrocyte proliferation, we used 2 hr BrdU labeling experiments on 3-week-old mice. Within the proliferative zone of the growth plates of wild-type mice 18% (±1.84%) of cells were labeled with BrdU, while only 14.8% (±1.95%) of cells were labeled in the growth plates of mice homozygous for *Comp* D469del, which corresponds to a decrease of 17% (*P* < 0.05) in the relative rates of chondrocyte proliferation in mutant growth plates (Fig. 4A). The relative levels of cell proliferation within growth plates of mice heterozygous for the mutation were comparable to those of the wild-type mice (data not shown).

To determine the cytotoxic effect of mutant *Comp* expression on chondrocyte viability, we performed the TUNEL assay on tibia growth plate sections from 3-week-old mice. In wild-type animals, TUNEL positive chondrocytes were observed primarily at the vascular invasion front and typically 1–2 positive cells were counted per section, whereas there were very few TUNEL positive cells in the hypertrophic zone and none at all observed in the proliferative zone of the growth plate (Fig. 4B; left panel). In contrast, there was a > fivefold increase (*P* < 0.0001) in TUNEL positive cells in the hypertrophic zone of mice homozygous for the mutation (Fig. 4C) and we also observed numerous clusters of TUNEL positive chondrocytes in the proliferative zone (Fig. 4B; right panel).

In order to identify the initial time of onset and subsequent progression of chondrocyte apoptosis, we also performed the TUNEL assay on the tibia growth plates of 5-day-old and 2-week-old mice that were wild-type and homozygous for *Comp* D469del (Supp. Fig. S5A). There was a ∼threefold increase in apoptosis in the proliferative zone of 5-day-old mice and a ∼fivefold increase in 2-week-old mice compared to wild-type littermates (Supp. Fig. S5B). There was no increase in apoptosis in the hypertrophic zone of 5-day-old mice, but a ∼twofold increase in 2-week-old mice compared to wild-type littermates (data not shown). Overall these data confirm that increased and spatially dysregulated apoptosis occurs shortly after birth and gets progressively worse, which is consistent with the gradual accumulation of mutant COMP and progressive shortening of the limbs.

### The Expression of Comp D469del Does Not Induce a Conventional Unfolded Protein Response (UPR)

Studies on *COMP* mutations to date have shown that mutant COMP is co-localized with chaperone proteins in the ER of chondrocytes from PSACH patients [Hecht et al., 2001; Vranka et al., 2001]. Furthermore, we have recently reported a UPR in mouse models of PSACH-MED harboring mutations in *Comp* (T585M) and *Matn3* (V194D) [Boot-Handford and Briggs, 2009; Leighton et al., 2007; Nundlall et al., 2010; Pirog-Garcia et al., 2007]. Therefore, in order to determine if the expression of *Comp* D469del caused a UPR, we looked at the relative expression of key chaperone proteins using a combination of real time PCR and SDS-PAGE Western blotting. qRT-PCR analysis showed no relative increase in the expression of Grp94 and Grp78/BiP at 5 days of age and Grp94, Grp78/BiP, Erp72, Cnx, and Crt at 3 weeks of age (Table 1). The corresponding protein levels of these chaperones was determined by Western blotting of intracellular proteins, which confirmed that there was no increase in the relative intracellular levels of Grp94, Grp78/BiP, and Erp72 in mutant chondrocytes at 3 weeks of age (Table 1). Furthermore, we could not detect any increase in the levels of phosphorylated eIF2α, a key downstream mediator of the UPR and there was also no increase in the relative expression of *Chop* at 5 days and 3 weeks of age (Table 1).

**Table 1:** The Expression Levels of Selected ER Stress and UPR-Associated Genes was Determined Using qRT-PCR Analysis of mRNA Isolated from Chondrocytes at 5 Days and 3 Weeks of Age and Western Blotting of Total Cartilage Protein Isolated from the Cartilage of 3-Week-Old Mice

	mRNA		Protein
		
ER stress/UPR marker	5 Days (wt vs. mut)	3 Week (wt vs. mut)	3 Week (wt vs. mut)
Grp94	0.57 (±0.17)	1.21 (± 0.32)	1.27 (±0.19)
Grp78/BiP	0.93 (±0.28)	1.20 (±0.18)	1.04 (±0.12)
Erp72/Pdia4	–	1.11 (±0.31)	1.31 (±0.18)
Calnexin	–	0.71 (±0.23)	–
Calreticulin	–	0.76 (±0.27)	–
CHOP	0.71 (±0.03)	0.91 (±0.35)	
eIF2α	–	–	1.06 (±0.31)
eIF2αP	–	–	0.98 (±0.09)

The table shows the relative levels of mRNA or protein in mutant samples compared to wild-type controls (wt vs. mut ± SEM). *n* ≥ 3 litters per genotype (∼5–8 mice per litter) and ≥3 separate experiments were performed in duplicate.

We also performed microarray analysis on RNA isolated from the rib chondrocytes of newborn and 5-day-old mice that were either heterozygous (5 day) or homozygous new born (newborn and 5 day) for *Comp* D469Del relative to the wild-type controls. These data confirmed that there was no increase in the relative expression of more than 250 genes associated with a conventional UPR (Supp. Table S1 and S2), some of which have previously been shown to be upregulated in chondrocytes from mice harboring the *Matn3* V194D mutation [Nundlall et al., 2010]. The only exception was fibroblast growth factor 18 (*Fgfr18*), which was upregulated twofold in newborn homozygous mutant mice. Finally, human HT1080 fibrosarcoma cells expressing the *COMP* p.D469del mutation, which showed the retention of mutant COMP protein (Supp. Fig. S6) also failed to demonstrate a conventional UPR (Supp. Table S2), thereby confirming consistency between relevant in vivo and in vitro models.

### Gene Expression Studies Show a Complex Disease Profile Implicating Oxidative Stress, Apoptosis, and Cell Cycle Arrest in the Pathogenesis of PSACH

Following the unequivocal exclusion of a classical UPR as a potential disease mechanism in *Comp* D469del mice, the Robust Multiarray Averaging (RMA) method was used to ascertain the top 20 significantly up- and downregulated genes in the chondrocytes from newborn and 5-day-old mice in order to identify potential genetic pathways involved in the pathogenesis of PSACH (Supp. Table S3–S6).

The genes upregulated in chondrocytes from newborn mice were clustered into the following groupings; oxidative stress (*Abcc9*, *Rgs5*, *Cdh5*), cell proliferation and/or apoptosis (*Igfbp3*, *Kera*, *Cilp*, *Lamb1-1*, *Sdpr*, *Sox17*), cell attachment and/or migration (*Ibsp*, *Chodl*, *Cd93*, *Cd34*, *Thbs4*, *Cdh5*, *Egfl6*), cell survival (*Ednrb*), and NF-κB signalling (*Meox2*) (Supp. Table S3). The most highly upregulated gene was *Igfbp3* (↑12.19), which has been shown to induce rapid apoptosis in a non-IGF-dependant manner and has antiproliferative effects via the transforming growth factor β (TGF-β) signalling pathway [Han et al., 2011; Lee et al., 2005]. The downregulated genes included those linked with NF-κB signalling (*Ccl5*, *Prdx2*, *T2bp*, *Hivep3, Tac1, Srxn1*), cell proliferation (*Pfkfb3*), cell survival (*Bdkrb1*), cell signalling (*Gng4*, *Lif*, *Otos, A2m, Rspo3*), and cell cycling (*Rnd1*) (Supp. Table S4). Interestingly, we found the downregulation of three members of the chemokine ligand protein family, namely *Ccl5*, *Cxcl2,* and *Cxcl16*, and also other genes that were involved in the same pathways such as *Hivep3* and *T2bp*.

By 5 days of age, the upregulated genes included those involved in oxidative stress and degradative pathways (*Amacr*, *Hmox1*, *Car3*, *Arsj*, *Ctsc*, *Heph*), cell survival and/or proliferation (*Ifi202b*, *Camk4*, *Sox11*, *Snca*, *Agtr2*), regulators of insulin receptor and IGFBP-3-dependent effects (*Enpp1*, *Lyve1*), and NF-κB signalling pathways (*Il1rl2*, *Phex*) (Supp. Table S5). The downregulated genes included those implicated in oxidative stress (*Npas4*, *Prdx2*), apoptosis (*Camk2d*, *Adi1*), cell cycle control (*Ddx3y*, *Eif2s3y*), cell proliferation/migration and cell–cell interactions (*Lypd3*, *Gjb4*, *Bop1*, *Cdh1*, *Hbegf*, *Trio*), and NF-κB signalling pathways (*Prdx2*, *Snno2*) (Supp. Table S6).

### The Downregulation of Peroxiredoxin-2 is a Consistent Finding in Both Mutant Mice and the Cell Culture Model

**Figure 5:**
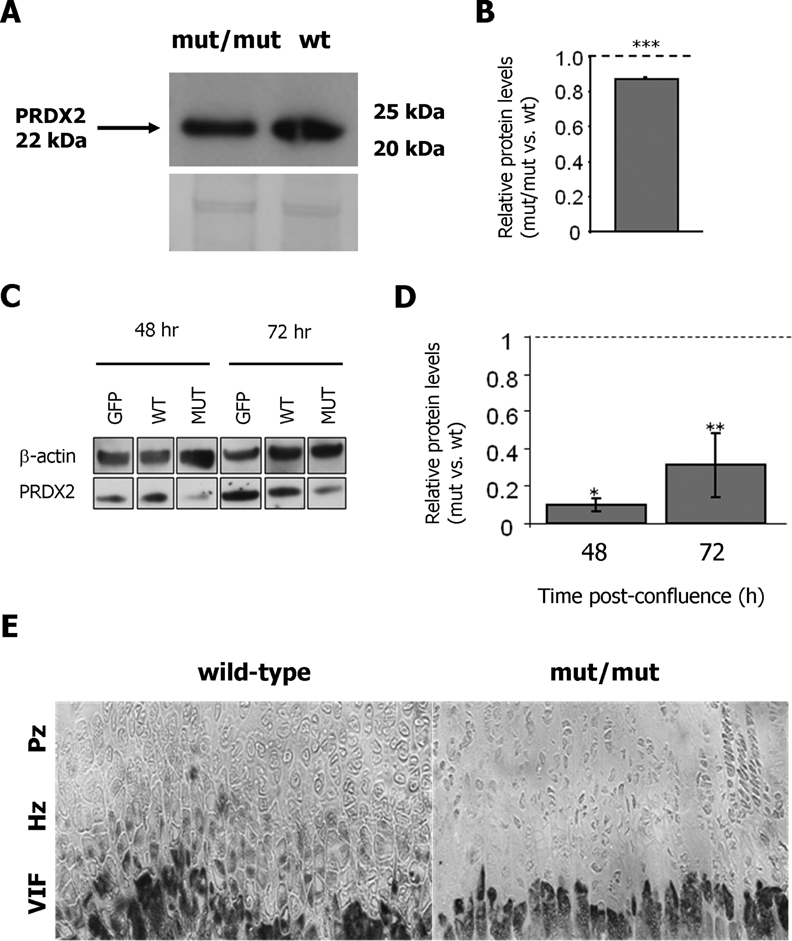
Peroxiredoxin 2 is consistently downregulated in mutant mice and a cell culture model of PSACH. **A:** Protein extracted from the xiphoid cartilage of 3-week-old wild-type (wt) and mutant (mut/mut) mice were analyzed by SDS-PAGE and Western blotting using an antibody against PRDX2 (∼22 kDa). **B:** Densitometry analysis on the Western blots confirmed that PRDX2 expression is reduced in the cartilage of mutant mice (mut/mut) compared to wild-type animals (wt). **C:** Cell lysates from HT1080 cells transfected with GFP alone (GFP), GFP-COMP wild-type (WT), and GFP-COMP mutant (MUT) were obtained either 48 or 72 hr after confluence and analyzed by SDS-PAGE and Western blotting using an antibody against PRDX2 (∼22 kDa) and normalized to β actin (∼42 kDa). **D:** Densitometry analysis on the Western blots confirmed that PRDX2 expression is reduced in cells transfected with the mutant protein compared to those transfected with the wild-type protein. **E:** Reduced PRDX2 expression in mutant mice cartilage was also confirmed by IHC on the tibial growth plates of 3-week-old mice. PRDX2 appears to be expressed by hypertrophic chondrocytes in the growth plate of wild-type mice, while its expression is much fainter in the same zone of the mutant mice growth plate. wt, wild-type mice; mut/mut, homozygous for D469del; VIF, vascular invasion front; Hz, hypertrophic zone; Pz, proliferative zone; *P < 0.05; **P < 0.005; ***P < 0.001.

Interestingly, the only gene whose expression was consistently changed at both time points was *Prdx2*, which was downregulated at both newborn (↓3.89) and 5 days of age (↓2.91) (Supp. Table S4 and S6). Furthermore, *PRDX2* expression was also significantly downregulated in the HT1080 cell culture model of COMP p.D469del (↓2.60) and at 5 days in mice heterozygous for the mutation (↓3.32) (data not shown). In view of these data and given the known association between the downregulation of *PRDX2* and apoptosis in human diseases such as Down syndrome and Fuchs Endothelial Dystrophy (FED) [Jurkunas et al., 2008; Sanchez-Font et al., 2003], we used a combination of Western blotting and IHC to validate the downregulation of peroxiredoxin-2 both in vivo and in vitro. Total protein was extracted from the cartilage of 3-week-old mice and used for SDS-PAGE and Western blotting (Fig. 5A). Following normalization by Ponceau staining for total protein, densitometry confirmed that the protein levels of peroxiredoxin-2 were significantly reduced by 13% (*P* < 0.0003) in mutant chondrocytes relative to wild-type (Fig. 5B). In addition, total cell lysate samples were collected from HT1080 cells at 48 and 72 hr after confluence and the relative levels of peroxiredoxin-2 were quantified by densitometry after normalizing to a β actin loading control (Fig. 5C). There was a >95% (*P* < 0.0001) decrease in the levels of peroxiredoxin-2 in the *COMP* p.D469del mutant cell line compared to wild-type (Fig. 5D). Finally, IHC was performed on the tibia growth plates of 3-week-old mice homozygous for the *Comp* D469del mutation and their wild-type littermates. This analysis demonstrated the intracellular staining of peroxiredoxin-2 in growth plate chondrocytes from mutant mice was much reduced in chondrocytes from wild-type mice growth plates (Fig. 5E).

## Discussion

A single mutation in *COMP*, the in-frame deletion of an aspartic acid codon (p.D469del) from the T3_7_ calcium-binding repeat of COMP, is responsible for approximately 30% of all PSACH [Hecht et al., 1995; Ikegawa et al., 1998; Kennedy et al., 2005a]. To determine the disease mechanisms that underpin the pathophysiology of PSACH in vivo, we generated a knock-in mouse model of PSACH resulting from this p.D469del mutation. This is the first physiologically relevant mouse model of PSACH to closely model the phenotypic and cellular consequences of *Comp* D469del. However, in contrast to the human PSACH phenotype, which is a dominant disease, both copies of the *Comp* D469del allele were required for the mice to develop a quantifiable chondrodysplasia phenotype. We have previously noted similar differences in other mice models of PSACH and MED, such as *Comp* Thr585Met [Pirog-Garcia et al., 2007] and *Matn3* Val194Asp [Leighton et al., 2007], and metaphyseal chondrodysplasia type Schmid *Col10a1* Asn617Lys [Rajpar et al., 2009]. Furthermore, a mouse model of achondroplasia showed a similar inconsistency [Li et al., 1999] and these discrepancies in phenotypic presentation are suggestive of dominance modification [Dixon and Dixon, 2004; Nadeau, 2001]

Mutant mice were normal at birth but developed significant short-limb dwarfism by 9 weeks of age (Fig. 1). This is consistent with the clinical progression of the PSACH phenotype in which patients are normal at birth but during childhood they exhibit progressive short-limbed dwarfism [Maroteaux and Lamy, 1959; Rimoin et al., 1994]. Mutant mice also developed a progressive hip dysplasia, which was characterized radiographically by a deflection from the vertical of the tuberosity of the ischium (Fig. 1). This observation was consistent with, although more severe than that reported in the *Comp* T585M [Pirog-Garcia et al., 2007], *Col10a1* N617K [Rajpar et al., 2009] and *Dtdst* A386V [Forlino et al., 2005] mutant mice. In contrast, neither a transgenic *Comp* D469Del mouse [Schmitz et al., 2008] nor the *Matn3* V194D mice [Leighton et al., 2007] had a recognizable hip dysplasia, even though the former displayed sternal malformations. This disparity in radiographic presentation in the transgenic *Comp* D469Del mouse may be due to differences in the level of transgene expression, while the *Matn3* V194D mutation may not have as pronounced an effect on the development of the pelvis.

The chondrocyte columns in the growth plates of mutant mice were disorganized and there were distinct changes to the morphology of individual chondrocytes. Immunohistochemical analysis also showed the retention of COMP, matrilin-3, and to a lesser extent type collagen IX, within the chondrocytes of mutant mice (Fig. 2). Numerous studies to date, both in vitro and in vivo, have consistently shown the accumulation of mutant COMP and since it interacts with both matrilin-3 and type IX collagen in vitro these interactions are possibly the cause of their co-retention. The effect that the retained mutant COMP has on the trafficking of type II collagen has not been fully resolved and there are conflicting reports as to whether type II collagen and other cartilage ECM molecules (such as aggrecan) are also retained in the rER of chondrocytes [Hecht et al., 2001; Vranka et al., 2001]. Our own IHC studies suggest that type II collagen is not co-retained with mutant COMP in the rER, however, there were clear differences in the localization of type II collagen in the ECM, which may be a direct consequence of the defect in trafficking of COMP, matrilin-3, and type IX collagen and/or the reduced levels of these proteins in the cartilage ECM (Supp. Fig. S4). Alternatively, the presence of mutant COMP in the ECM may disrupt specific interactions, thereby influencing in turn the localization of its binding partners. This hypothesis is consistent with the pericellular localization of mutant COMP and wild-type matrilin-3 in the resting zone of mutant growth plates (Fig. 2D and F).

Quite striking were the regions of hypocellularity within the proliferative zone (Fig. 2), which were consistent with the significantly increased and dysregulated apoptosis within this zone of the growth plate. This finding is in agreement with studies on cultured PSACH chondrocytes, which show an increase in cell death following prolonged culture in vitro [Duke et al., 2003], and also a transgenic model of *Comp* D469del in which there was a significant increase in cell death when it was crossed onto a *Comp* null background [Schmitz et al., 2008]. However, in this transgenic model the increase in apoptotic cells was primarily detected at the interface with the secondary center of ossification, which is in direct contrast to our knock-in model in which increased apoptosis is seen in the proliferative and hypertrophic zones. It is difficult to explain this difference in the localization of apoptosis, but it is possible that differences in the relative levels of mutant *Comp* expression between these two models might influence the location of dysregulated apoptosis.

Based on our recent findings in the PSACH and MED mouse models of *Comp* T585M [Pirog-Garcia et al., 2007] and *Matn3* V194D [Leighton et al., 2007; Nundlall et al., 2010] mutations, we hypothesized that the expression of *Comp* D469del would cause ER stress and a UPR. However, qRT-PCR and Western blot analysis both failed to reveal any increases in the relative expression of key chaperone proteins at 5 days and 3 weeks of age (Table 1), which was confirmed by extensive microarray studies performed at newborn and 5 days of age (Supp. Table S1 and S2). This is the first study to unequivocally establish the relative expression levels of chaperone proteins in PSACH chondrocytes and to establish that there is no conventional UPR due to the expression of mutant COMP D469del. Previous studies using patient cartilage or cultured patient chondrocytes have shown that mutant COMP is associated with various chaperone proteins, such as CRT, PDI, Grp94, Erp72, and Grp78/BiP, in distended cisternae of the rER, both in vivo and in vitro [Hecht et al., 2001; Vranka et al., 2001]. Furthermore, immunoprecipitation followed by Western blotting of proteins from control chondrocytes demonstrated that CRT, Grp94, and Erp72 were directly associated with normal COMP, which was confirmed by Flourescence Resonance Energy Transfer (FRET) [Hecht et al., 2001]. However, all of the previous studies on patient samples did not determine quantitatively the relative expression levels of these chaperone proteins and it is likely that their localization to the ER, and association with mutant COMP and other retained ECM proteins, is an indirect result of protein accumulation in the ER and the fact that they are all ER resident proteins. This observation is supported by IHC staining for PDI, BiP and Grp78, which show no differences in the localization and relative amounts of these three chaperones in mice that are wild-type or mutant for D469del COMP (Supp. Fig. S7). Finally, we also determined that there was no increased phosphorylation of eIF2α, a key mediator of the UPR, or any increase in the relative expression of CHOP, an important apoptosis-associated downstream target of prolonged UPR (Table 1). Taken together these data suggest that the expression of mutant COMP is inducing apoptosis that is independent of a conventional UPR.

A consistent finding in all three knock-in mouse models of PSACH and MED is a significant reduction in chondrocyte proliferation and the increased and/or spatially dysregulated apoptosis in the cartilage growth plate [Leighton et al., 2007; Pirog-Garcia et al., 2007]. The effects of the *Comp* D469del mutation on chondrocyte proliferation and apoptosis was more pronounced than in our previously published models, which is consistent with the more severe phenotype caused by the *COMP* p.D469del mutation in PSACH patients. These detrimental alterations to chondrocyte proliferation and survival are therefore likely to be key factors in the initiation and progression of PSACH and MED. However, the specific genetic pathways leading to reduced proliferation and apoptosis are clearly different depending on the mutated gene and/or the specific mutation. For example, our previous studies have demonstrated that the expression of *Matn3* V194D and *Comp* T585M cause ER stress that results in a conventional UPR characterized by the upregulation of various chaperone proteins [Leighton et al., 2007; Nundlall et al., 2010; Pirog-Garcia et al., 2007], which is clearly not the case for *Comp* D469del (Supp. Table S1 and S2).

In the case of *Comp* T585M, the UPR appears sufficient for the correct folding and secretion of mutant COMP, however, either the UPR itself or the antimorphic effect of mutant COMP in the ECM initiates CHOP-mediated apoptosis [Pirog-Garcia et al., 2007]. In contrast, the UPR is not capable of mediating the correct folding and secretion of the majority of mutant matrilin-3 (V194D), which appears to accumulate in the rER of chondrocytes and eventually leading to dysregulated apoptosis [Leighton et al., 2007]. However, unlike *Comp* T585M, this is not CHOP-mediated and the specific genetic pathways that result in apoptosis remain unresolved [Nundlall et al., 2010]. Finally, we show in this study that the expression of *Comp* D469del results in significantly increased apoptosis that is independent of both a conventional UPR and CHOP-mediated apoptosis. It is interesting to note that the *COMP* p. T585M mutation is generally accepted to result in a phenotype that is defined as either mild PSACH or severe MED [Briggs et al., 1998; Song et al., 2003], in contrast to the p.D469del mutation that always causes the most severe form of PSACH. This difference in cellular mechanisms of *COMP* mutations provides insight into the broad clinical spectrum of the PSACH-MED disease group.

Our microarray studies performed at newborn and 5 days of age have demonstrated that there are changes in the expression levels of a variety of different genes involved in oxidative stress, cell survival, attachment and/or migration, cell cycle arrest and apoptosis, and NF-κB signalling. These gene expression changes are consistent with the detrimental changes in chondrocyte phenotype that we have observed in the growth plate (i.e., reduced proliferation and increased apoptosis) and likely characterize a novel form of chondrocyte stress that is key to the pathogenesis of PSACH in this mouse model.

Interestingly, several recent studies have shown that UPR-independent pathways, such as the ER overload response (EOR), are associated with the aggregation of mutant proteins in the ER, which induce a toxic gain of function. This is particularly the case with the serpinopathies in which the aggregation of insoluble misfolded α_1_-antitrypsin triggers an alternative ER stress response that is independent of the UPR and involves the activation of NF-κB signalling [Ekeowa et al., 2010]. Recently, a role for *Prdx2* in reducing apoptosis through NF-κB signalling has been demonstrated in mouse granulose cells [Yang et al., 2011], while the downregulation of PRDX2 is implicated angiotensis II-mediated podocyte apoptosis [Hsu et al., 2011], thus providing further evidence that these pathways are important for cell survival. In addition, we have shown that the most highly upregulated gene in newborn chondrocytes was *Igfbp3*, while other genes (i.e., *Lyve1*), known to be mediators of *Igfbp3*-dependant effects, were upregulated at 5 days of age. These data suggest that *Igfbp3*-mediated apoptosis may play a role in the initiation and progression of chondrocyte apoptosis due to the expression and accumulation of COMP D469del.

In summary, we have generated a new mouse model of PSACH and demonstrated that expression of the most common form of mutant COMP causes a novel type of chondrocyte stress that is independent of a conventional UPR. This interesting finding highlights the emerging field of UPR-independent pathways and supports alternative pathways such as the EOR in the pathogenesis of human genetic diseases.
