# Supplementary material for: A Novel Form of Chondrocyte Stress is Triggered by a COMP Mutation Causing Pseudoachondroplasia
Source: Hum Mutat. 2011 Oct 17;33(1):218–31. doi: 10.1002/humu.21631 (PMC3320758; doi:10.1002/humu.21631)
Supplement: Supplementary file 1 [file humu0033-0218-SD1.pdf]

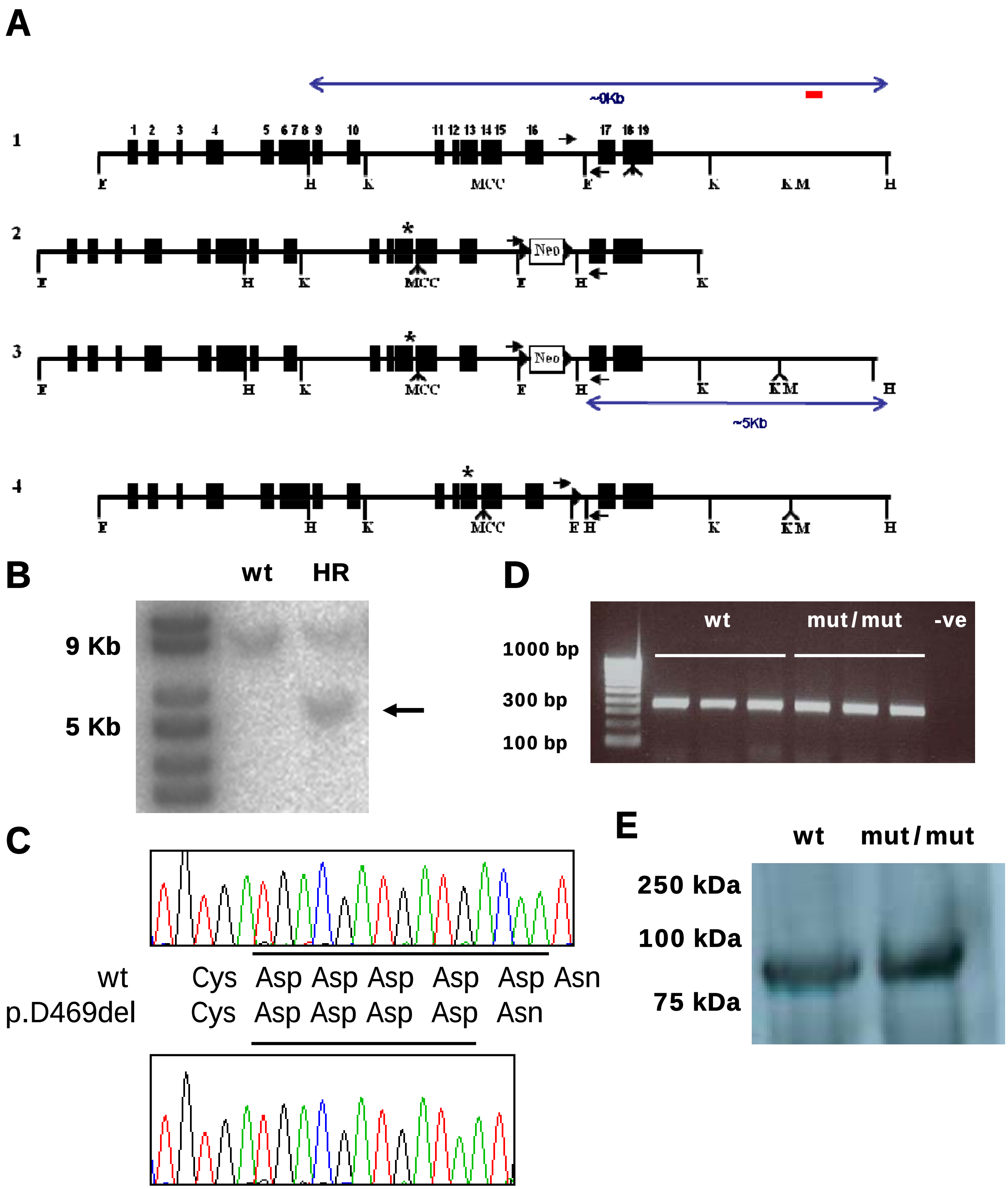

Supp. Figure S1. Targeting strategy to generate the Comp D469del PSACH mouse. **A)** 1) The genomic organisation of *Comp* showing the location of key restriction sites and the external probe (red bar). 2) The targeting construct with a *loxP* (arrowheads) flanked *Neo* cassette cloned into intron 16 of *Comp*. An asterisk marks the site of the introduced mutation in exon 13 of *Comp*. 3) Modified *Comp* locus following homologous recombination. The external probe detects the two *Hind* III restriction fragments (blue arrows). 4) The recombinant *Comp* allele following *Cre*-mediated recombination. Restriction enzyme sites

are:- E – *Eco* RI, H – *Hind* III, K – *Kpn* I, M – *Mlu* I, C – *Cla* I. **B)** Southern Blot showing wild-type (wt) and recombinant (HR) ES clones following *Hind* III digestion. **C)** DNA sequencing confirming that the mutation is present in the recombinant alleles. **D**) Agarose gel electrophoresis of PCR amplified cDNA confirming a lack of genomic DNA contamination. The correct cDNA fragments at ~350 bp were observed whilst there were no bands at ~700 bp corresponding to genomic DNA. **E)** SDS-PAGE and Western blot showing comparable levels of COMP protein in wild-type and mutant cartilage at 3 weeks of age. Key:- wt (wild-type), m/m (mice homozygous for the mutation).

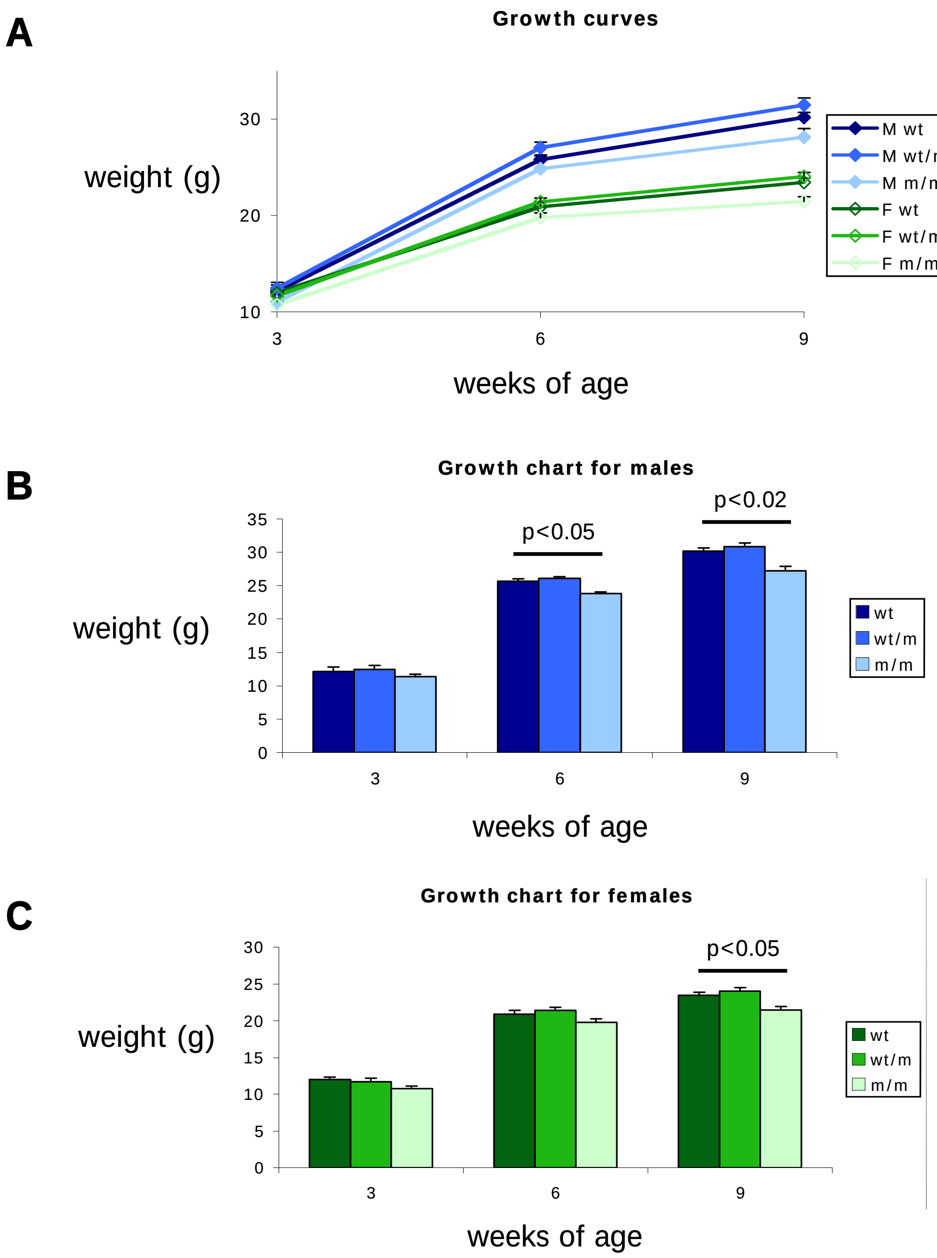

Supp. Figure S2. Growth curves for mice over a 9-week time period. Male and female mice were weighed at 3, 6 and 9 weeks after birth and their weights charted. **A)** Male and female mice homozygous for *Comp*:ΔD469 mutation had slower growth curves than wild type and heterozygous mutant animals. Separate growth charts for males and females are shown at 3, 6 and 9 weeks for mice of all three genotypes. **B)** Homozygous *Comp* p.D469del males were significantly smaller than their litter-mates at 6 weeks and 9 weeks of age. **C)** Homozygous *Comp* p.D469del females were significantly smaller than their littermates by 9 weeks of age. N=7 mice per sex per genotype, One-Way ANOVA.

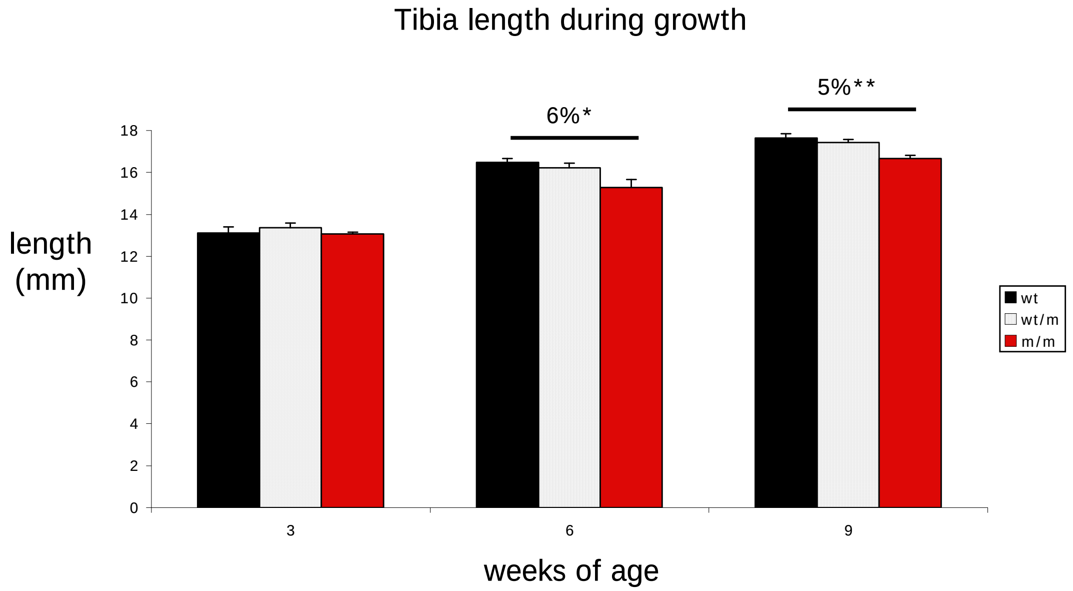

Supp. Figure S3. Measurement of tibia length from male mice between 3 and 9 weeks of age. Tibias lengths of male mice were measured at 3, 6 and 9 weeks of age for wild type, heterozygous and homozygous *Comp* p.D469del littermates. Tibias from mice homozygous for *Comp* p.D469del were significantly shorter that their littermates at 6 and 9 weeks of age. N>7 mice per genotype, One-way ANOVA .

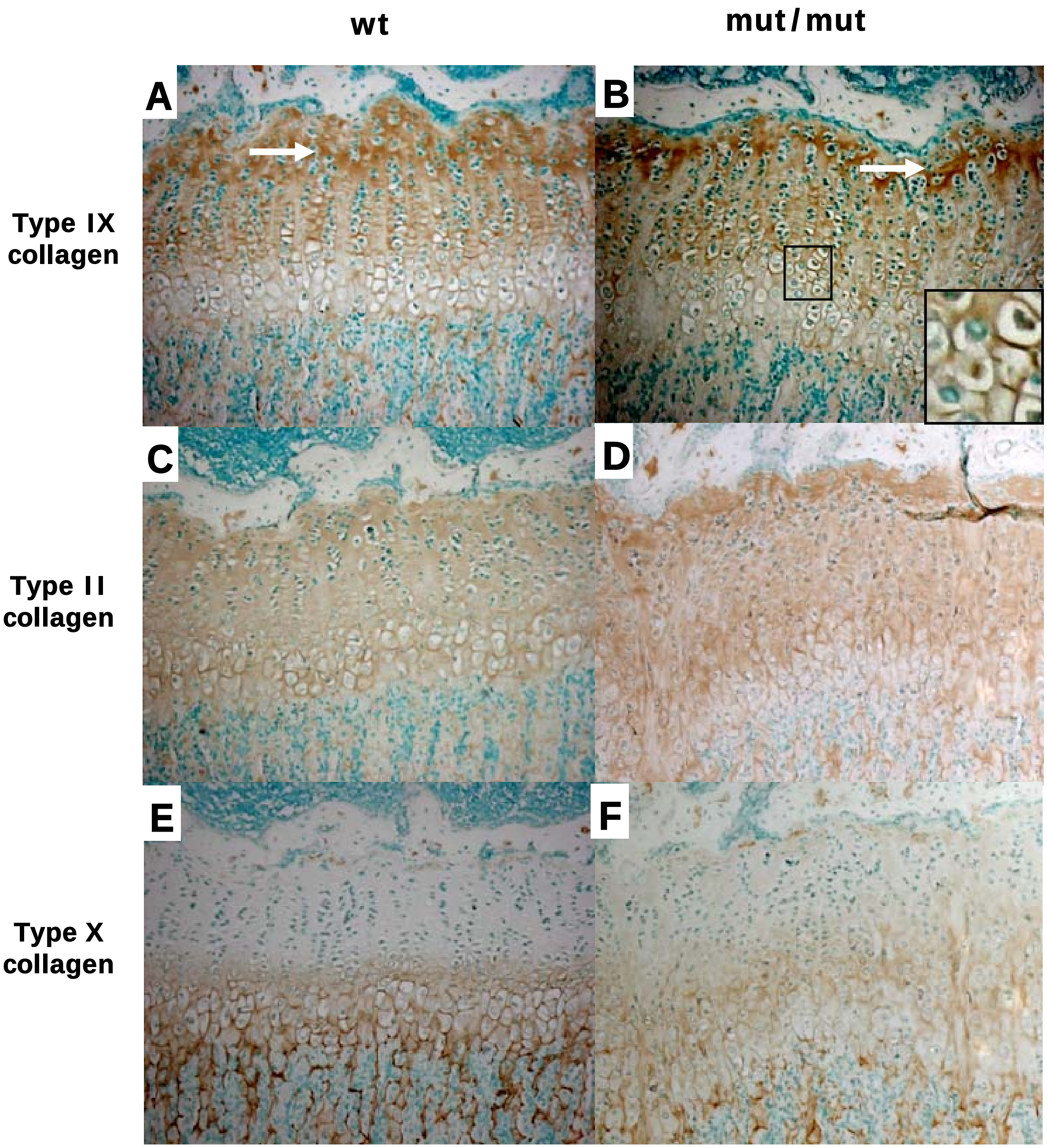

Supp. Figure S4. Representative images of wild type (wt) and mutant (mut/mut) growth plates showing the localisation of cartilage collagen proteins. **A & B**) IHC using a type IX collagen antibody showed some intracellular retention in mutant chondrocytes (**insert**), whilst staining to type IX collagen was more pronounced in the resting zone (**white arrow**) and in the pericellular region of the early proliferative zones of both wild type and mutant growth plates. **C & D**) IHC using a type II collagen antibody demonstrated patchy mis-localisation in the mutant growth plate, whilst type X collagen (**E & F**) was also diffuse in the growth plate of mice homozygous for p.D469del.

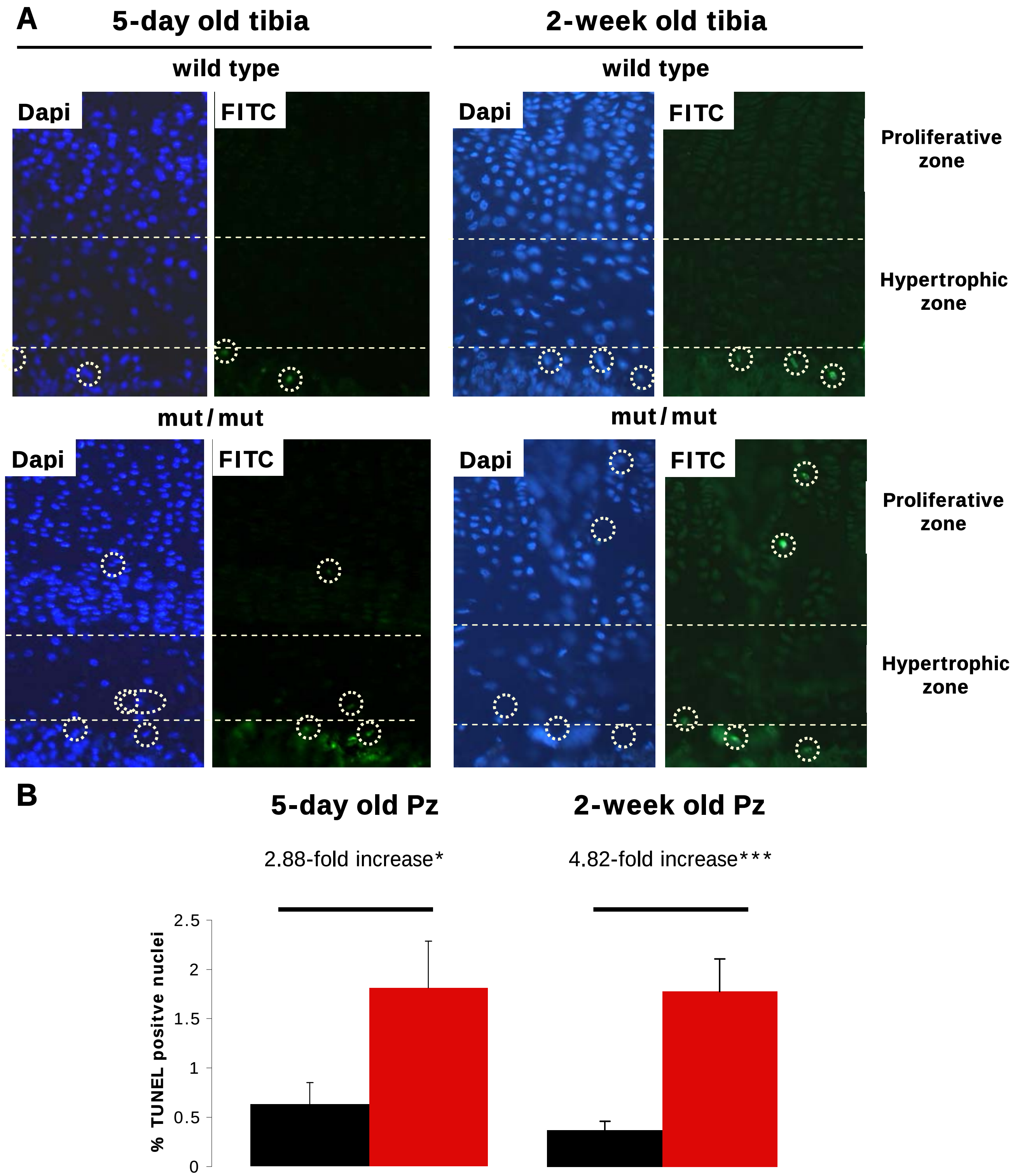

Supp. Figure S5. Time of onset and progression of apoptosis in the growth plate of *Comp* D469del mice. **A)** TUNEL positive cells (white circles) were detected in the proliferative zones of growth plates from mice homozygous for the p.D469del COMP mutation, but not in their wild-type littermates. **B)** Quantification of the TUNEL assay results showed a significant increase in apoptosis in the proliferative zone of growth plates from mice homozygous for the mutation (n=36 section from 3 mice per genotype, independent samples t-test, * $p<0.05$, ***$p<0.001$).

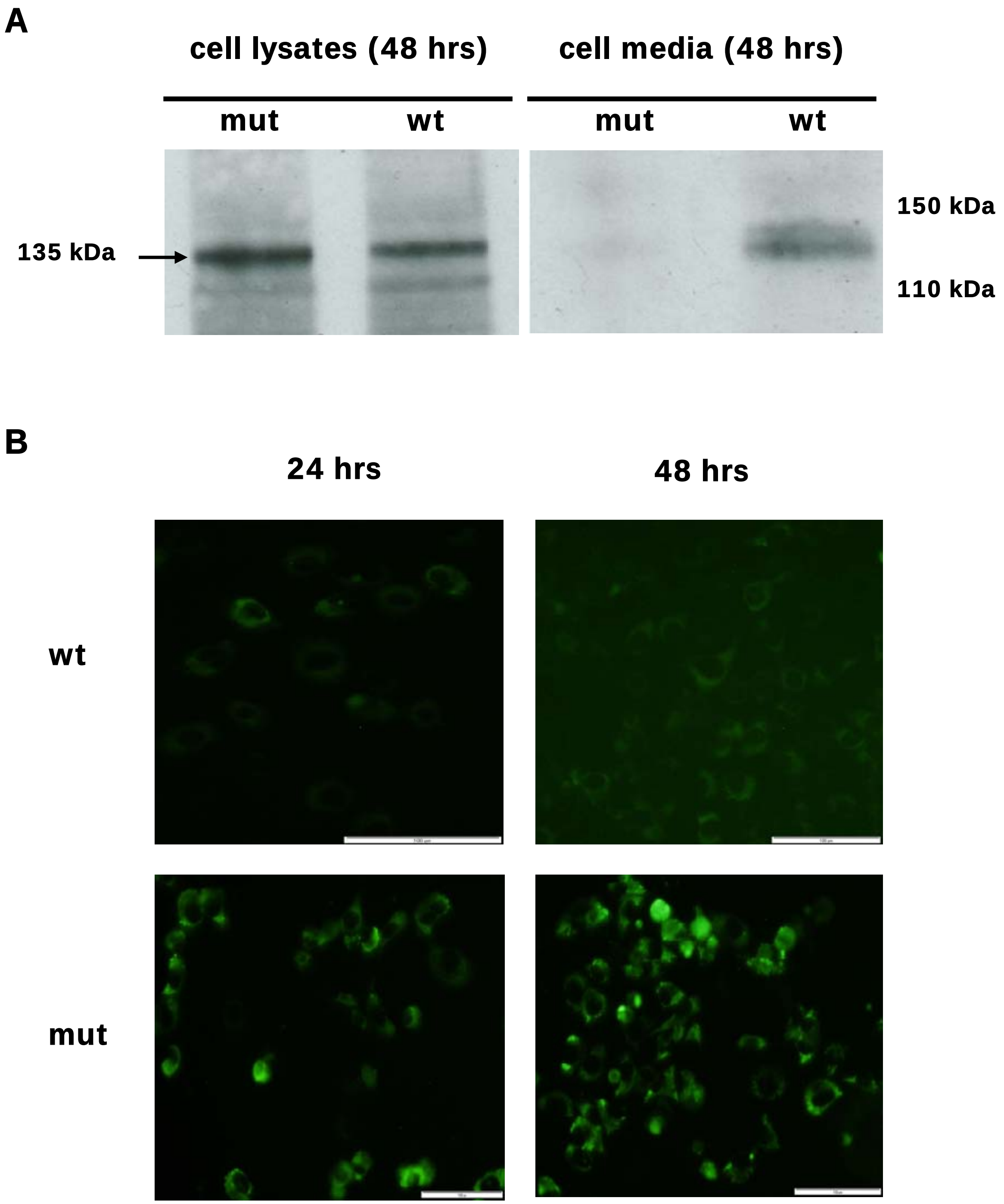

Supp. Figure S6. HT1080 cells show retention of mutant COMP. **A**) Cell lysate and medium samples were collected at 48 hr post confluence from wild-type and mutant COMP-GFP cell lines. Approximately 20 µg of protein was resolved by 4-10% SDS-PAGE and Western blotted with a COMP specific antibody (diluted 1:500, Genetex). A ~135 kDa band was present in the media from the wild-type cell line which was absent in the mutant cell line. **B**) HT1080 cells expressing recombinant wild-type and mutant p.D469del COMP-GFP fusion protein were grown in cell culture medium and analysed under fluorescent microscope at 24 and 48 hrs after cells were seeded (357.1 exposure time). Cells harboring p.D469del COMP mutation show intercellular localisation of mutant protein. Scale bar 100µm. Key: wt (wild-type); mut (mutant).

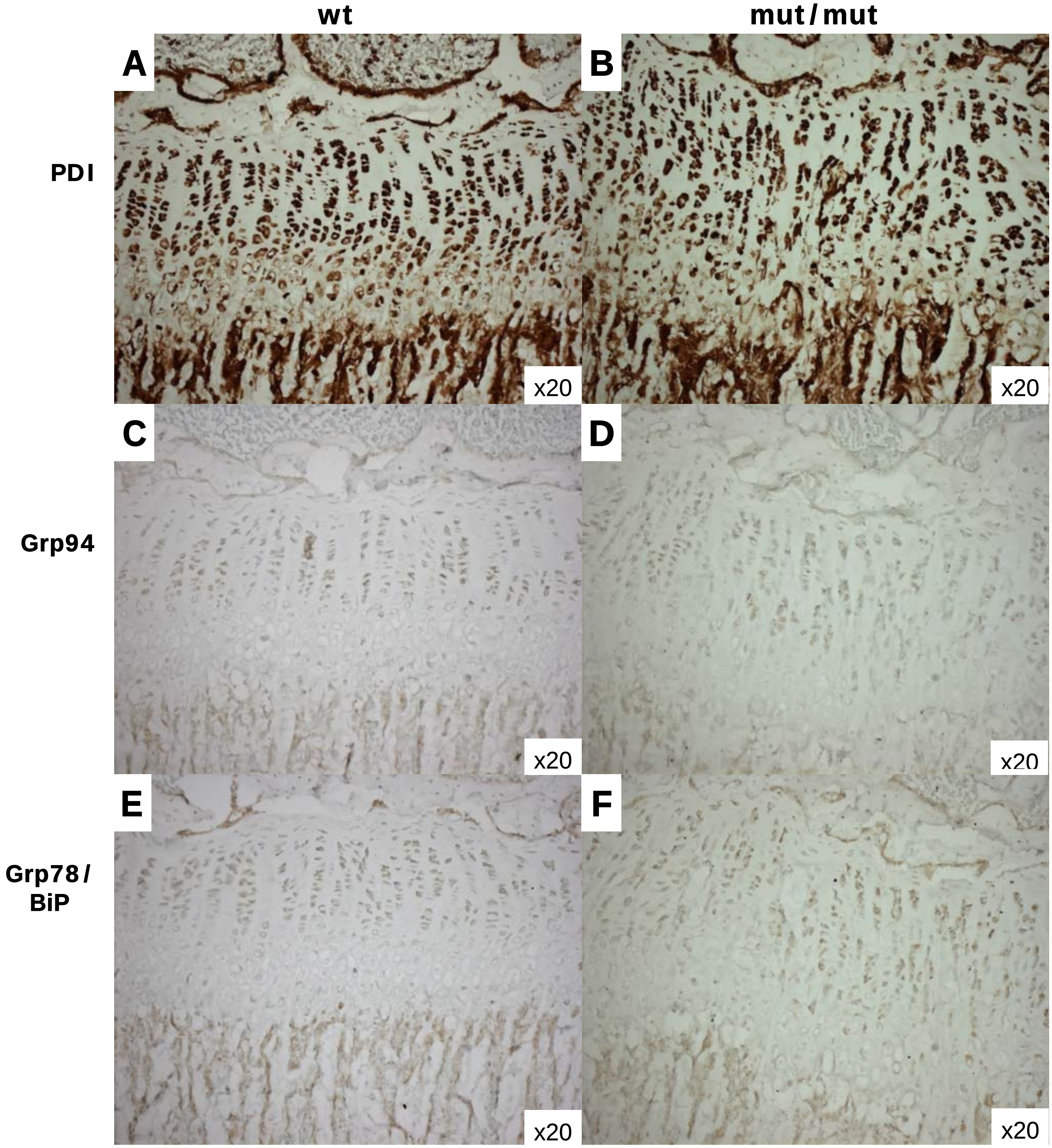

Supp. Figure S7. Representative images of wild type (wt) and mutant (mut/mut) growth plates showing the localisation of ER resident chaperone proteins. **A & B**) IHC using an antibody against PDI **C & D**) IHC using an antibody against Grp94. **E & F**) IHC using an antibody against Grp78/BiP.

Supp. Table S1. The fold change in the relative expression levels of UPR, ERAD and cartilage genes in homozygous *Comp* p.D469del mice at new born and 5-days of age

RNA was isolated the rib chondrocytes of new born and 5 day-old wild-type and *Comp* p.D469del$^{m/m}$ mutant mice. The relative expression levels of genes calculated using the Robust Multiarray Averaging (RMA) method are shown with the significance value given in parentheses. Highlighted in yellow are values that reach a statistical significance difference compared to wild-type (i.e. P<0.05). The gene order and categorization of the genes is based on that published by Cameron et al PLoS ONE (*in press*).

The classical receptors of endoplasmic reticulum stress			**Genotype**	
NCBI	Gene Name	Gene Symbol	**D469del m/m NB**	**D469del m/m 5D**
NM_001081304	Activating transcription factor 6	*Atf6*	1.14 (0.433)	1.13 (0.972)
NM_023913	Endoplasmic reticulum to nucleus signalling 1	*Ern1*	-1.21 (0.256)	-1.05 (0.999)
NM_010121	Eukaryotic translation initiation factor 2 alpha kinase 3	*Eif2ak3*	1.17 (0.432)	1.11 (0.999)
The downstream genes of the ER stress receptor pathways				
NCBI	Gene Name	*Gene Symbol*		
Atf6-related:-				
NM_009722	ATPase, Ca++ transporting, cardiac muscle, slow twitch 2	*Atp2a2*	1.20 (0.428)	-1.06 (0.999)
NM_007837	DNA-damage inducible transcript 3	*Ddit3*	-1.20 (0.368)	1.16 (0.875)
NM_022310	Heat shock protein 5	*Hspa5*	1.17 (0.499)	1.11 (0.999)
NM_027400	Lectin, mannose-binding, 1	*Lman1*	1.40 (0.289)	1.12 (0.999)
NM_029103	Mesencephalic astrocyte-derived neurotrophic factor	*Manf*	1.43 (0.210)	1.13 (0.999)
NM_008725	Natriuretic protein precursor type A	*Nppa*	-1.14 (0.272)	-1.03 (0.999)
NM_011850	Nuclear receptor subfamily 0, group b, member 2	*Nr0b2*	-1.07 (0.512)	-1.02 (0.999)
NM_008814	Pancreatic and duodenal homeobox 1	*Pdx1*	-1.26 (0.170)	-1.01 (0.999)
NM_028769	Synovial apoptosis inhibitor 1 (Synoviolin)	*Syvn1*	1.11 (0.422)	-1.15 (0.996)
NM_020275	Tumor necrosis factor receptor superfamily, member 10b	*Tnfrsf10b*	-1.86 (0.112)	-1.22 (0.836))
NM_013842	X-box binding protein 1	*Xbp1*	1.05 (0.640)	1.01 (0.999)
Eif2ak3-related:-				
NM_007498	Activating transcription factor 3	*Atf3*	-1.21 (0.493)	-1.01 (0.999)
NM_007837	DNA-damage inducible transcript 3	*Ddit3*	-1.19 (0.368)	1.16 (0.933)
NM_001005509	Eukaryotic translation initiation factor 2a	*Eif2a*	1.05 (0.559)	1.10 (0.999)
NM_026114	Eukaryotic translation initiation factor 2, subunit 1 alpha	*Eif2s1*	-1.21 (0.357)	1.07 (0.999)
NM_023913	Endoplasmic reticulum to nucleus signalling 1	*Ern1*	-1.21 (0.256)	-1.05 (0.999)
NM_022310	Heat shock protein 5	*Hspa5*	1.17 (0.499)	1.11 (0.999)

NM_010481	Heat shock protein 9	*Hspa9*	-1.10 (0.532)	1.05 (0.999)
NM_010512	Insulin-like growth factor 1	*Igf1*	1.24 (0.567)	2.02 (0.495)
NM_028782	lon peptidase 1, mitochondrial	*Lonp1*	-1.04 (0.999)	-1.11 (0.610)
NM_010902	Nuclear factor, erythroid derived 2, like 2	*Nfe2l2*	-1.01 (0.691)	1.14 (0.922)
NM_008689	Nuclear factor of light polypeptide gene enhancer in B-cells 1	*Nfkb1*	-1.91 (0.138)	-1.17 (0.999)
NM_019408	Nuclear factor of light polypeptide gene enhancer in B-cells 2	*Nfkb2*	-1.47 (0.209)	-1.17 (0.999)
NM_008654	Protein phosphatase 1, regulatory (inhibitor) subunit 15a	*Ppp1r15a*	-1.16 (0.442)	-1.19 (0.912)
NM_001039521	Rrn3 RNA polymerase I transcription factor homolog (yeast)	*Rrn3*	-1.01 (0.999)	-1.17 (0.689)
NM_011716	Wolfram syndrome 1 homolog	*Wfs1*	-1.21 (0.414)	-1.04 (0.999)
Ern1-related:-				
NM_001081304	Activating transcription factor 6	*Atf6*	1.14 (0.433)	1.13 (0.972)
NM_007837	DNA-damage inducible transcript 3	*Ddit3*	-1.20 (0.368)	1.16 (0.836)
NM_022310	Heat shock protein 5	*Hspa5*	1.17 (0.499)	1.11 (0.999)
NM_011952	Mitogen-activated protein kinase 3	*Mapk3*	1.10 (0.535)	-1.11 (0.999)
NM_016700	Mitogen-activated protein kinase 8	*Mapk8*	1.15 (0.468)	-1.04 (0.999)
NM_008689	Nuclear factor of light polypeptide gene enhancer in B-cells 1	*Nfkb1*	-1.91 (0.138)	-1.17 (0.999)
NM_019408	Nuclear factor of light polypeptide gene enhancer in B-cells 2	*Nfkb2*	-1.47 (0.209)	-1.17 (0.999)
NM_015760	NADPH oxidase 4	*Nox4*	2.21 (0.57)	1.58 (0.656)
NM_011951	Mitogen-activated protein kinase 14	*Mapk14*	1.03 (0.394)	1.03 (0.999)
NM_028769	Synovial apoptosis inhibitor 1 (Synoviolin)	*Syvn1*	1.11 (0.422)	-1.15 (0.996)
NM_013693	Tumor necrosis factor	*Tnf*	-1.29 (0.112)	1.06 (0.999)
NM_020275	Tumor necrosis factor receptor superfamily, member 10b	*Tnfrsf10b*	-1.86 (0.112)	-1.22 (0.791)
NM_011716	Wolfram syndrome 1 homolog	*Wfs1*	-1.21 (0.414)	-1.04 (0.999)
NM_013842	X-box binding protein 1	*Xbp1*	1.05 (0.640)	1.01 (0.999)
Additional ER stress sensors				
NCBI	Gene Name	*Gene Symbol*		
NM_030080	Androgen induced bZIP transcription factor	*Aibzip*	-1.12 (0.421)	1.22 (0.999)
NM_178661	BBF2 human homolog on chromosome 7	*Bbf2h7*	1.04 (0.643)	-1.16 (0.999)
NM_145365	cAMP responsive element binding protein 3-like 3	*Crebh*	1.03 (0.657)	1.00 (0.999)
NM_013497	cAMP responsive element binding 3	*Luman*	1.16 (0.177)	1.06 (0.999)
NM_011957	Old astrocyte specifically induced substance	*Oasis*	2.01 (0.139)	-1.04 (0.999)
Endoplasmic reticulum associated degradation (ERAD) components				
NCBI	Gene Name	*Gene Symbol*		

NM_007597	Calnexin	*Canx*	1.12 (0.506)	1.22 (0.972)
NM_007591	Calreticulin	*Calr*	1.20 (0.413)	1.09 (0.999)
NM_024207	Der1-like domain family, member 1 (Derlin 1)	*Derl1*	-1.06 (0.576)	1.04 (0.999)
NM_033562	Der1-like domain family, member 2 (Derlin 2)	*Derl2*	-1.08 (0.541)	1.11 (0.949)
NM_024440	Der1-like domain family, member 3 (Derlin 3)	*Derl3*	-1.04 (0.619)	-1.04 (0.999)
NM_010072	Dolichol-phosphate (beta-D) mannosyltransferase 1	*Dpm1*	1.17 (0.407)	1.13 (0.999)
NM_010073	Dolichol-phosphate (beta-D) mannosyltransferase 2	*Dpm2*	-1.09 (0.487)	-1.04 (0.999)
NM_138677	ER degradation enhancer, mannosidase alpha-like 1	*Edem1*	1.18 (0.376)	-1.02 (0.999)
NM_145537	ER degradation enhancer, mannosidase alpha-like 2	*Edem2*	1.08 (0.292)	1.04 (0.999)
NM_001039644	ER degradation enhancer, mannosidase alpha-like 3	*Edem3*	1.57 (0.183)	1.09 (0.999)
NM_022331	Homocysteine-inducible, endoplasmic reticulum stress-inducible, ubiquitin-like domain member 1	*Herpud1*	1.33 (0.248)	1.04 (0.999)
NM_020586	HERPUD family member 2	*Herpud2*	1.06 (0.415)	1.31 (0.988)
NM_007952	Protein disulphide isomerase associated 3	*Pdia3*	1.38 (0.264)	1.12 (0.965)
NM_009787	Protein disulphide isomerase associated 4	*Pdia4*	-1.08 (0.604)	1.76 (0.407)
NM_028295	Protein disulphide isomerase associated 5	*Pdia5*	1.22 (0.325)	1.07 (0.999)
NM_027959	Protein disulphide isomerase associated 6	*Pdia6*	1.31 (0.196)	-1.09 (0.999)
NM_016906	Sec61 alpha subunit 1	*Sec61a1*	1.22 (0.477)	1.12 (0.974)
NM_021305	Sec61 alpha subunit 2	*Sec61a2*	1.08 (0.585)	1.03 (0.999)
NM_008408	STT3, subunit of the oligosaccharyltransferase complex, homolog A	*Stt3a*	1.02 (0.558)	1.13 (0.996)
NM_024222	STT3, subunit of the oligosaccharyltransferase complex, homolog A	*Stt3b*	1.05 (0.575)	1.15 (0.999)
NM_028769	Synovial apoptosis inhibitor 1 (Synoviolin)	*Syvn1*	1.11 (0.422)	-1.15 (0.996)
Proteasomal subunits:				
NCBI	Gene Name	*Gene Symbol*		
NM_011965	Proteasome (prosome, macropain) subunit, alpha type 1	*Psma1*	1.05 (0.611)	1.12 (0.999)
NM_008944	Proteasome (prosome, macropain) subunit, alpha type 2	*Psma2*	1.13 (0.419)	1.06 (0.999)
NM_011184	Proteasome (prosome, macropain) subunit, alpha type 3	*Psma3*	1.01 (0.686)	1.03 (0.999)
NM_011966	Proteasome (prosome, macropain) subunit, alpha type 4	*Psma4*	-1.10 (0.523)	1.04 (0.999)
NM_011967	Proteasome (prosome, macropain) subunit, alpha type 5	*Psma5*	1.17 (0.398)	1.07 (0.999)
NM_011968	Proteasome (prosome, macropain) subunit, alpha type 6	*Psma6*	1.20 (0.234)	-1.03 (0.999)
NM_011969	Proteasome (prosome, macropain) subunit, alpha type 7	*Psma7*	1.10 (0.529)	1.05 (0.999)
AK010717	Proteasome (prosome, macropain) subunit, alpha type 8	*Psma8*	-1.03 (0.647)	-1.05 (0.999)
NM_011185	Proteasome (prosome, macropain) subunit, beta type 1	*Psmb1*	-1.03 (0.656)	1.08 (0.999)
NM_011970	Proteasome (prosome, macropain) subunit, beta type 2	*Psmb2*	1.06 (0.593)	1.05 (0.999)
NM_011971	Proteasome (prosome, macropain) subunit, beta type 3	*Psmb3*	-1.03 (0.662)	-1.06 (0.999)

NM_008945	Proteasome (prosome, macropain) subunit, beta type 4	*Psmb4*	1.02 (0.602)	-1.02 (0.999)
NM_011186	Proteasome (prosome, macropain) subunit, beta type 5	*Psmb5*	-1.06 (0.581)	-1.07 (0.999)
NM_008946	Proteasome (prosome, macropain) subunit, beta type 6	*Psmb6*	1.30 (0.151)	1.08 (0.999)
NM_011187	Proteasome (prosome, macropain) subunit, beta type 7	*Psmb7*	-1.03 (0.647)	1.14 (0.999)
NM_010724	Proteasome (prosome, macropain) subunit, beta type 8	*Psmb8*	-1.03 (0.647)	-1.05 (0.999)
NM_013585	Proteasome (prosome, macropain) subunit, beta type 9	*Psmb9*	1.12 (0.562)	-1.05 (0.999)
NM_013640	Proteasome (prosome, macropain) subunit, beta type 10	*Psmb10*	1.22 (0.370)	1.05 (0.999)
NM_008947	Protease (prosome, macropain) 26S subunit, ATPase 1	*Psmc1*	-1.50 (0.051)	-1.04 (0.999)
NM_011188	Protease (prosome, macropain) 26S subunit, ATPase 2	*Psmc2*	1.08 (0.552)	1.11 (0.999)
NM_008948	Protease (prosome, macropain) 26S subunit, ATPase 3	*Psmc3*	1.09 (0.484)	1.05 (0.999)
NM_011874	Protease (prosome, macropain) 26S subunit, ATPase 4	*Psmc4*	-1.02 (0.656)	-1.08 (0.999)
NM_008950	Protease (prosome, macropain) 26S subunit, ATPase 5	*Psmc5*	-1.15 (0.379)	1.03 (0.999)
NM_025959	Protease (prosome, macropain) 26S subunit, ATPase 6	*Psmc6*	1.06 (0.601)	1.08 (0.999)
NM_027357	Proteasome (prosome, macropain) 26S subunit, non-ATPase, 1	*Psmd1*	1.06 (0.353)	-1.05 (0.999)
NM_134101	Proteasome (prosome, macropain) 26S subunit, non-ATPase, 2	*Psmd2*	1.00 (0.999)	1.10 (0.700)
NM_009439	Proteasome (prosome, macropain) 26S subunit, non-ATPase, 3	*Psmd3*	1.06 (0.979)	1.02 (0.999)
NM_008951	Proteasome (prosome, macropain) 26S subunit, non-ATPase, 4	*Psmd4*	1.15 (0.368)	-1.02 (0.999)
NM_080554	Proteasome (prosome, macropain) 26S subunit, non-ATPase, 5	*Psmd5*	1.07 (0.537)	1.07 (0.999)
NM_025550	Proteasome (prosome, macropain) 26S subunit, non-ATPase, 6	*Psmd6*	1.05 (0.579)	-1.01 (0.999)
NM_010817	Proteasome (prosome, macropain) 26S subunit, non-ATPase, 7	*Psmd7*	1.02 (0.999)	1.06 (0.664)
NM_026545	Proteasome (prosome, macropain) 26S subunit, non-ATPase, 8	*Psmd8*	1.04 (0.628)	-1.02 (0.999)
NM_026000	Proteasome (prosome, macropain) 26S subunit, non-ATPase, 9	*Psmd9*	1.02 (0.999)	-1.07 (0.941)
NM_016883	Proteasome (prosome, macropain) 26S subunit, non-ATPase, 10	*Psmd10*	1.05 (0.999)	1.11 (0.998)
NM_178616	Proteasome (prosome, macropain) 26S subunit, non-ATPase, 11	*Psmd11*	-1.04 (0.604)	-1.04 (0.999)
NM_025894	Proteasome (prosome, macropain) 26S subunit, non-ATPase, 12	*Psmd12*	-1.04 (0.999)	1.05 (0.999)
NM_011875	Proteasome (prosome, macropain) 26S subunit, non-ATPase, 13	*Psmd13*	1.03 (0.999)	1.04 (0.999)
NM_021526	Proteasome (prosome, macropain) 26S subunit, non-ATPase, 14	*Psmd14*	-1.07 (0.999)	-1.07 (0.999)
NM_011189	Proteasome (prosome, macropain) 28S subunit, alpha	*Psme1*	1.27 (0.187)	1.04 (0.999)
NM_011192	Proteasome (prosome, macropain) 28S subunit, 3	*Psme3*	-1.22 (0.380)	1.00 (0.999)
NM_134013	Proteasome (prosome, macropain) 28S subunit, 4	*Psme4*	1.06 (0.350)	1.05 (0.999)
NM_212446	Proteasome (prosome, macropain) inhibitor subunit 1	*Psmf1*	-1.17 (0.382)	-1.05 (0.999)
Individual molecular chaperones:				
NCBI	Gene Name	*Gene Symbol*		
NM_008298	DnaJ (Hsp40) homolog, subfamily A, member 1	*Dnaja1*	1.09 (0.662)	-1.06 (0.999)
NM_019794	DnaJ (Hsp40) homolog, subfamily A, member 2	*Dnaja2*	1.04 (0.999)	-1.12 (0.746)
NM_023646	DnaJ (Hsp40) homolog, subfamily A, member 3	*Dnaja3*	-1.25 (0.275)	1.01 (0.999)

NM_021422	DnaJ (Hsp40) homolog, subfamily A, member 4	*Dnaja4*	-1.24 (0.427)	-1.53 (0.973)
NM_018808	DnaJ (Hsp40) homolog, subfamily B, member 1	*Dnajb1*	-1.33 (0.319)	-1.65 (0.855)
NM_008299	DnaJ (Hsp40) homolog, subfamily B, member 3	*Dnajb3*	1.08 (0.930)	-1.07 (0.999)
NM_025926	DnaJ (Hsp40) homolog, subfamily B, member 4	*Dnajb4*	1.11 (0.778)	-1.08 (0.999)
NM_019874	DnaJ (Hsp40) homolog, subfamily B, member 5	*Dnajb5*	-1.10 (0.934)	-1.20 (0.942)
NM_001037940	DnaJ (Hsp40) homolog, subfamily B, member 6	*Dnajb6*	-1.09 (0.918)	1.13 (0.927)
NM_021317	DnaJ (Hsp40) homolog, subfamily B, member 7	*Dnajb7*	-1.02 (0.847)	1.04 (0.999)
NM_019964	DnaJ (Hsp40) homolog, subfamily B, member 8	*Dnajb8*	1.03 (0.999)	-1.18 (0.963)
NM_013760	DnaJ (Hsp40) homolog, subfamily B, member 9	*Dnajb9*	-1.08 (0.557)	1.03 (0.999)
NM_020266	DnaJ (Hsp40) homolog, subfamily B, member 10	*Dnajb10*	-1.63 (0.081)	-1.35 (0.734)
NM_026400	DnaJ (Hsp40) homolog, subfamily B, member 11	*Dnajb11*	1.06 (0.954)	1.12 (0.999)
NM_019965	DnaJ (Hsp40) homolog, subfamily B, member 12	*Dnajb12*	1.06 (0.895)	-1.02 (0.999)
NM_007869	DnaJ (Hsp40) homolog, subfamily C, member 1	*Dnajc1*	-1.11 (0.936)	1.15 (0.999)
NM_009584	DnaJ (Hsp40) homolog, subfamily C, member 2	*Dnajc2*	-1.03 (0.941)	1.06 (0.999)
NM_008929	DnaJ (Hsp40) homolog, subfamily C, member 3B	*Dnajc3a (p58IPK)*	1.16 (0.824)	1.27 (0.810)
NM_020566	DnaJ (Hsp40) homolog, subfamily C, member 4	*Dnajc4*	-1.20 (0.221)	-1.12 (0.999)
NM_016775	DnaJ (Hsp40) homolog, subfamily C, member 5	*Dnajc5*	1.07 (0.999)	1.00 (0.999)
NM_025489	DnaJ (Hsp40) homolog, subfamily C, member 5 beta	*Dnajc5b*	-1.02 (0.471)	-1.07 (0.491)
NM_177677	DnaJ (Hsp40) homolog, subfamily C, member 5 gamma	*Dnajc5g*	-1.08 (0.524)	1.06 (0.999)
NM_198412	DnaJ (Hsp40) homolog, subfamily C, member 6	*Dnajc6*	1.03 (0.983)	1.01 (0.999)
NM_019795	DnaJ (Hsp40) homolog, subfamily C, member 7	*Dnajc7*	-1.17 (0.944)	1.07 (0.999)
NM_172400	DnaJ (Hsp40) homolog, subfamily C, member 8	*Dnajc8*	1.06 (0.964)	1.05 (0.999)
NM_134081	DnaJ (Hsp40) homolog, subfamily C, member 9	*Dnajc9*	-1.07 (0.977)	1.25 (0.763)
NM_024181	DnaJ (Hsp40) homolog, subfamily C, member 10	*Dnajc10*	1.06 (0.863)	1.02 (0.999)
NM_172704	DnaJ (Hsp40) homolog, subfamily C, member 11	*Dnajc11*	-1.15 (0.698)	1.01 (0.999)
NM_013888	DnaJ (Hsp40) homolog, subfamily C, member 12	*Dnajc12*	1.01 (0.866)	-1.05 (0.999)
NM_001163026	DnaJ (Hsp40) homolog, subfamily C, member 13	*Dnajc13*	1.11 (0.591)	1.08 (0.999)
NM_028873	DnaJ (Hsp40) homolog, subfamily C, member 14	*Dnajc14*	1.28 (0.523)	1.26 (0.734)
NM_025384	DnaJ (Hsp40) homolog, subfamily C, member 15	*Dnajc15*	-1.12 (0.897)	-1.11 (0.943)
NM_172338	DnaJ (Hsp40) homolog, subfamily C, member 16	*Dnajc16*	1.01 (0.999)	1.02 (0.999)
NM_139139	DnaJ (Hsp40) homolog, subfamily C, member 17	*Dnajc17*	-1.05 (0.993)	-1.03 (0.999)
NM_029669	DnaJ (Hsp40) homolog, subfamily C, member 18	*Dnajc18*	1.09 (0.832)	1.12 (0.999)
NM_026332	DnaJ (Hsp40) homolog, subfamily C, member 19	*Dnajc19*	-1.13 (0.537)	1.11 (0.999)
NM_010479	Heat shock protein 1A	*Hspa1a*	-1.24 (0.793)	-1.05 (0.999)
NM_013558	Heat shock protein 1-like	*Hspa1l*	-1.03 (0.999)	-1.10 (0.954)
NM_008301	Heat shock protein 2	*Hspa2*	1.02 (0.999)	-1.46 (0.681)
NM_008300	Heat shock protein 4	*Hspa4*	-1.03 (0.999)	-1.10 (0.954)
NM_011020	Heat shock protein 4 like	*Hspa4l*	1.60 (0.062)	1.03 (0.999)
NM_022310	Heat shock protein 5	*Hspa5*	1.17 (0.499)	1.11 (0.999)

NM_031165	Heat shock protein 8	*Hspa8*	-1.04 (0.999)	-1.16 (0.976)
NM_010481	Heat shock protein 9	*Hspa9*	-1.10 (0.684)	1.05 (0.999)
NM_175199	Heat shock protein 12A	*Hspa12a*	1.02 (0.963)	-1.07 (0.999)
NM_028306	Heat shock protein 12B	*Hspa12b*	1.39 (0.291)	-1.02 (0.999)
NM_015765	Heat shock protein 14	*Hspa14*	1.00 (0.999)	1.12 (0.999)
NM_013560	Heat shock protein 1	*Hspb1*	1.06 (0.954)	-1.06 (0.999)
NM_024441	Heat shock protein 2	*Hspb2*	-1.01 (0.999)	-1.28 (0.690)
NM_019960	Heat shock protein 3	*Hspb3*	-1.66 (0.188)	1.21 (0.861)
NM_001012401	Heat shock protein, alpha-crystallin-related, B6	*Hspb6*	-1.28 (0.489)	1.00 (0.999)
NM_013868	Heat shock protein family, member 7 (cardiovascular)	*Hspb7*	1.13 (0.852)	-1.02 (0.999)
NM_030704	Heat shock protein 8 (Hspb8)	*Hspb8*	1.07 (0.999)	-1.32 (0.818)
NM_175111	Hspb associated protein 1	*Hspbap1*	-1.14 (0.523)	-1.05 (0.999)
NM_025486	Heat shock protein c171	*Hspc171*	-1.02 (0.999)	-1.01 (0.999)
NM_010477	Heat shock protein 1 (chaperonin)	*Hspd1*	-1.14 (0.304)	-1.06 (0.999)
NM_010480	Heat shock protein 90kDa alpha (cytosolic), class A member 1	*Hsp90aa1*	-1.04 (0.986)	-1.05 (0.999)
NM_008302	Heat shock protein 90kDa alpha (cytosolic), class B member 1	*Hsp90ab1*	1.10 (0.529)	-1.06 (0.999)
NM_011631	Heat shock protein 90kDa beta (Grp94), member 1	*Hsp90b1 (Grp94)*	1.26 (0.721)	1.26 (0.902)
NM_013559	Heat shock protein 110	*Hsp110*	-1.36 (0.328)	-1.19 (0.997)
Cartilage genes:				
NCBI	Gene Name	*Gene Symbol*		
NM_007424	Aggrecan	*Agc1*	1.00 (0.999)	-1.12 (0.993)
NM_007614	Catenin (cadherin associated protein), beta 1	*Ctnnb1*	1.21 (0.725)	1.14 (0.999)
NM_031163	Collagen, type II, alpha 1	*Col2a1*	-1.03 (0.999)	1.02 (0.999)
NM_009933	Collagen, type VI, alpha 1	*Col6a1*	1.19 (0.671)	-1.03 (0.999)
NM_146007	Collagen, type VI, alpha 2	*Col6a2*	1.44 (0.224)	-1.02 (0.999)
XM_897036	Collagen, type VI, alpha 3	*Col6a3*	1.35 (0.129)	-1.02 (0.999)
NM_007739	Collagen, type VIII, alpha 1	*Col8a1*	1.65 (0.366)	1.32 (0.999)
NM_199473	Collagen, type VIII, alpha 2	*Col8a2*	1.52 (0.354)	1.82 (0.635)
NM_007740	Collagen, type IX, alpha 1	*Col9a1*	-1.02 (0.999)	1.05 (0.999)
NM_007741	Collagen, type IX, alpha 2	*Col9a2*	-1.42 (0.316)	1.00 (0.999)
NM_009936	Collagen, type IX, alpha 3	*Col9a3*	-1.24 (0.573)	-1.06 (0.999)
NM_007729	Collagen, type XI, alpha 1	*Col11a1*	-1.01 (0.999)	1.03 (0.999)
NM_009926	Collagen, type XI, alpha 2	*Col11a2*	-1.12 (0.628)	1.04 (0.999)
NM_016685	Cartilage oligomeric matrix protein	*Comp*	1.06 (0.999)	1.01 (0.999)
NM_010217	Connective tissue growth factor	*Ctgf*	1.30 (0.360)	-1.04 (0.999)

NM_010056	Distal-less homeobox 5	*Dlx5*	1.12 (0.622)	1.03 (0.999)
NM_008005	Fibroblast growth factor 18	*Fgf18*	2.44 (0.005)	-1.33 (0.974)
NM_008010	Fibroblast growth factor receptor 3	*Fgfr3*	-1.13 (0.952)	1.04 (0.999)
NM_013500	Hyaluronan and proteoglycan link protein 1	*Hapln1*	1.03 (0.999)	-1.06 (0.999)
NM_010544	Indian hedgehog	*Ihh*	-1.03 (0.992)	-1.09 (0.999)
NM_010769	Matrilin 1	*Matn1*	1.49 (0.663)	-1.39 (0.828)
NM_016762	Matrilin 2	*Matn2*	1.36 (0.711)	1.19 (0.999)
NM_010770	Matrilin 3	*Matn3*	-1.09 (0.962)	-1.02 (0.999)
NM_013592	Matrilin 4	*Matn4*	1.04 (0.999)	1.11 (0.999)
NM_008970	Parathyroid hormone-like peptide	*Pthlh*	1.85 (0.055)	-1.01 (0.999)
NM_011199	Parathyroid hormone 1 receptor	*Pthr1*	-1.19 (0.413)	-1.01 (0.999)
NM_022017	Transient receptor potential cation channel, subfamily V, member 4	*Trpv4*	-1.08 (0.979)	-1.23 (0.957)
Hypertrophic cartilage genes:				
NM_007431	Alkaline phosphatase, liver/bone/kidney	*Akp2*	1.81 (0.333)	-1.14 (0.999)
NM_009925	Collagen, type X, alpha 1	*Col10a1*	1.01 (0.999)	-1.49 (0.801)
NM_013599	Matrix metallopeptidase 9	*Mmp9*	1.15 (0.835)	1.00 (0.999)
NM_008607	Matrix metallopeptidase 13	*Mmp13*	1.50 (0.398)	1.53 (0.734)
NM_008608	Matrix metallopeptidase 14	*Mmp14*	1.60 (0.493)	1.14 (0.999)
NM_009263	Secreted phosphoprotein 1	*Spp1*	1.89 (0.070)	1.67 (0.970)
NM_009820	Runt related transcription factor 2	*Runx2*	1.18 (0.510)	1.05 (0.999)
Classical apoptosis related genes:				
NCBI	Gene Name	*Gene Symbol*		
NM_007527	Bcl2-associated X protein	*Bax*	1.23 (0.523)	-1.04 (0.999)
NM_009741	B-cell leukemia/lymphoma 2	*Bcl2*	-1.13 (0.943)	1.01 (0.999)
NM_009808	Caspase 12	*Casp12*	1.43 (0.779)	1.46 (0.987)
NM_009810	Caspase 3	*Casp3*	1.03 (0.949)	1.04 (0.999)
NM_007611	Caspase 7	*Casp7*	1.32 (0.222)	1.06 (0.999)
NM_015733	Caspase 9	*Casp9*	-1.08 (0.994)	-1.05 (0.999)
NM_007837	DNA damage inducible transcript 3	*Ddit3*	-1.19 (0.368)	1.16 (0.999)
NM_015774	ERO1-like (S. cerevisiae)	*Ero1l*	1.18 (0.963)	-1.03 (0.999)
NM_026184	ERO1-like beta (S. cerevisiae)	*Ero1lb*	1.01 (0.999)	-1.01 (0.999)
NM_146120	Gelsolin	*Gsn*	1.05 (0.906)	-1.11 (0.999)
NM_010705	Lectin, galactose binding, soluble 3	*Lgals3*	1.10 (0.665)	1.00 (0.999)
NM_008580	Mitogen-activated protein kinase kinase kinase 5	*Map3k5*	-1.15 (0.505)	-1.01 (0.999)
NM_016700	Mitogen-activated protein kinase 8	*Mapk8*	1.15 (0.687(	-1.03 (0.999)

NM_008689	Nuclear factor of kappa light polypeptide gene enhancer in B-cells 1, p105	*Nfkb1*	-1.92 (0.138)	-1.17 (0.999)
NM_019408	Nuclear factor of kappa light polypeptide gene enhancer in B-cells 2, p49/p100	*Nfkb2*	-1.47 (0.308)	-1.17 (0.999)
NM_008654	Protein phosphatase 1, regulatory (inhibitor) subunit 15A	*Ppp1r15a*	-1.16 (0.442)	-1.19 (0.999)
NM_009422	TNF receptor-associated factor 2	*Traf2*	-1.25 (0.301)	1.07 (0.999)
NM_175093	Tribbles homolog 3 (Drosophila)	*Trib3*	-1.40 (0.498)	1.27 (0.907)
NM_207680	Bcl2-like 11 (apoptosis facilitator)	*Bcl2l11*	1.30 (0.597)	-1.14 (0.999)
NM_133234	Bcl2 binding component 3	*Bbc3*	-1.19 (0.914)	-1.08 (0.999)
NM_026929	Chac, cation transport-like regulator 1 (E.coli)	*Chac1*	-1.51 (0.247)	1.26 (0.999)
NM_019816	Apoptosis antagonizing transcription factor	*Aatf*	-1.08 (0.902)	1.07 (0.999)
NM_213659	Signal transducer and activator of transcription 3	*Stat3*	-1.13 (0.853)	1.08 (0.999)
NM_011716	Wolfram syndrome 1 homolog (human)	*Wfs1*	-1.21 (0.414)	-1.04 (0.999)
NM_009652	Thymoma viral proto-oncogene 1	*Akt1*	1.01 (0.690)	1.02 (0.999)

Supp. Table S2. The fold change in the relative expression levels of selected conventional UPR and/or ERAD genes in heterozygous *Comp* p.D469del mice and *COMP* p.D469del cell culture model

RNA was isolated the rib chondrocytes of 5 day-old wild-type and mutant mice (*Comp* p.D469del$^{m/m}$ and *Comp* p.D469del$^{wt/mut}$) or HT1080 cells stably transfected with control or *COMP* p.D469del cDNA (cells were harvested 48 hrs post confluence). The relative expression levels of various UPR associated genes calculated using the PUMA method are shown with the significance value given in parentheses. Highlighted in yellow are values that reach a statistically significance difference compared to wild-type (i.e. P>0.95 for up regulated genes and P<0.05 for down regulated genes. The gene order and categorization of the genes is based on that published by Cameron et al PLoS ONE (*in press*).

Classical endoplasmic reticulum stress receptors and targets				
NCBI designation	Gene Name	Gene Symbol	**D469del wt/m 5D**	**D469del cells**
NM_023913	Endoplasmic reticulum to nucleus signalling 1	Ern1	1.21 (0.436)	-1.06 (0.549)
NM_010121	Eukaryotic translation initiation factor 2 alpha kinase 3	Eif2ak3	2.44 (0.593)	6.22 (0.540)
NM_007837	DNA-damage inducible transcript 3	Ddit3	-1.73 (0.003)	1.30 (0.690)
NM_022310	Heat shock protein 5	Hspa5/BiP*	-1.56 (0.122)	1.42 (0.953)
NM_013842	X-box binding protein 1	Xbp1	-1.11 (0.333)	1.21 (0.982)
Selected genes involved with a classical UPR and/or ERAD				
NCBI designation	Gene Name	Gene Symbol		
NM_007597	Calnexin	Canx	-1.06 (0.348)	1.22 (0.847)
NM_007591	Calreticulin	Calr*	-1.08 (2.7E-3)	1.47 (0.920)
NM_024207	Der1-like domain family, member 1 (Derlin 1)	Derl1	1.11 (0.693)	1.16 (0.773)
NM_033562	Der1-like domain family, member 2 (Derlin 2)	Derl2	1.07 (0.614)	1.39 (0.927)
NM_024440	Der1-like domain family, member 3 (Derlin 3)	Derl3	-1.53 (0.269)	2.27 (0.762)
NM_021422	DnaJ (Hsp40) homolog, subfamily A, member 4	Dnaja4	-2.02 (0.011)	-2.09 (0.288)
NM_019964	DnaJ (Hsp40) homolog, subfamily B, member 8	Dnajb8	-1.13 (0.448)	-1.77 (0.190)
NM_013760	DnaJ (Hsp40) homolog, subfamily B, member 9	Dnajb9	-1.11 (0.287)	-1.10 (0.352)
NM_020266	DnaJ (Hsp40) homolog, subfamily B, member 10	Dnajb10	-1.19 (0.094)	-1.78 (0.148)
NM_019965	DnaJ (Hsp40) homolog, subfamily B, member 12	Dnajb12	1.38 (0.908)	-1.05 (0.487)
NM_007869	DnaJ (Hsp40) homolog, subfamily C, member 1	Dnajc1	-1.11 (0.313)	1.07 (0.659)
NM_020566	DnaJ (Hsp40) homolog, subfamily C, member 4	Dnajc4	1.18 (0.707)	-1.31 (0.352)
NM_025489	DnaJ (Hsp40) homolog, subfamily C, member 5 beta	Dnajc5b	2.00 (0.801)	1.56 (0.699)
NM_019795	DnaJ (Hsp40) homolog, subfamily C, member 7	Dnajc7	1.10 (0.716)	1.18 (0.736)
NM_013888	DnaJ (Hsp40) homolog, subfamily C, member 12	Dnajc12	1.00 (0.505)	-5.90 (0.235)
NM_025384	DnaJ (Hsp40) homolog, subfamily C, member 15	Dnajc15	1.32 (0.997)	1.19 (0.813)
NM_138677	ER degradation enhancer, mannosidase alpha-like 1	Edem1	1.28 (0.874)	-1.07 (0.384)
NM_145537	ER degradation enhancer, mannosidase alpha-like 2	Edem2	-2.09 (0.376)	-1.07 (0.328)
NM_001039644	ER degradation enhancer, mannosidase alpha-like 3	Edem3	1.18 (0.737)	1.19 (0.185)

NM_022331	Homocysteine-inducible, endoplasmic reticulum stress-inducible, ubiquitin-like domain member 1	Herpud1	-1.17 (0.140)	1.48 (0.999)
NM_020586	Homocysteine-inducible, endoplasmic reticulum stress-inducible, ubiquitin-like domain member 2	Herpud2	1.25 (0.438)	-1.03 (0.447)
NM_013558	Heat shock protein 1-like	Hspa1l	1.30 (0.602)	1.20 (0.550)
NM_011020	Heat shock protein 4 like	Hspa4l	-1.19 (0.365)	4.12 (1)
NM_015765	Heat shock protein 14	Hspa14	-1.13 (0.401)	1.64 (0.998)
NM_013560	Heat shock protein 1	Hspb1	-1.75 (5.1E-05)	-2.54 (0.336)
NM_019960	Heat shock protein 3	Hspb3	-1.86 (0.298)	1.20 (0.533)
NM_025486	Heat shock protein c171	Hspc171	-1.12 (0.205)	1.20 (0.828)
NM_011631	Heat shock protein 90kDa beta (Grp94), member 1	Hsp90b1 (Grp94)	-1.52 (4.9E-3)	-1.96 (0.517)
NM_029103	Mesencephalic astrocyte-derived neurotrophic factor	Manf/Armet	-1.33 (0.011)	1.14 (0.859)
NM_007952	Protein disulphide isomerase associated 3	Pdia3	-1.03 (0.475)	-1.21 (0.250)
NM_009787	Protein disulphide isomerase associated 4	Pdia4	-1.27 (0.073)	-1.18 (0.267)
NM_028295	Protein disulphide isomerase associated 5	Pdia5	-1.14 (0.246)	1.15 (0.734)
NM_027959	Protein disulphide isomerase associated 6	Pdia6	-1.27 (0.171)	1.14 (0.862)

Supp. Table S3. The top 20 most significantly up regulated genes in chondrocytes from new born *Comp* p.D469del$^{m/m}$ mutant mice relative to wild type

Gene ID	Gene Name	Gene Symbol	Fold change	Putative role in PSACH pathology
AV175389	Insulin-like growth factor binding protein 3	*Igfbp3*	12.19	IGFBP3 induces **rapid apoptosis** in a non-IGF-dependant manner and has **anti-proliferative effects** via transforming growth factor β (TGF-β) signalling pathway.
NM_008438	Keratocan	*Kera*	11.00	An ECM protein and member of the SLRPs. Plays a role in regulating cellular processes such as **proliferation** and modulation of osteoprogenitor lineage differentiation.
NM_008218	Hemoglobin alpha, adult chain 1 and 2	*Hba-a1* *Hba-a2*	10.11	Involved in oxygen transport from the lung to various peripheral tissues.
L20232	Integrin binding sialoprotein	*Ibsp*	9.97	A member of the small integrin-binding ligand N-linked glycoprotein (SIBLING) family. Binds tightly to hydroxyapatite and appears to form an integral part of the mineralized matrix. Important for cell-matrix interactions and directly promotes Arg-Gly-Asp-dependent **cell attachment** (see also *Egfl6*).
BG791642	ATP-binding cassette, sub-family C (CFTR/MRP), member 9	*Abcc9*	9.25	Subunit of ATP-sensitive potassium channels (KATP). Mitochondrial ATP-sensitive potassium channels can **inhibit apoptosis** induced by **oxidative stress**.
BE630003	Cartilage intermediate layer protein, nucleotide pyrophosphohydrolase	*Cilp*	9.17	Cilp has the ability to suppress IGF1-induced **proliferation** and sulfated proteoglycan synthesis and inhibits ligand-induced IGF1R auto phosphorylation. May inhibit TGFB1-mediated induction of cartilage matrix genes via its interaction with TGFB1. Over expression may **impair chondrocyte growth** and matrix repair.
BG970109	Laminin B1 subunit 1	*Lamb1-1*	9.05	Binding to cells via a high affinity receptor, laminin is thought to mediate the attachment, migration and organization of cells into tissues during embryonic development by interacting with other extracellular matrix components. Cell surface expression induces **apoptosis**.
AF311699	Chondrolectin	*Chodl*	8.65	Important for **cell migration** and cell-cell interactions.
BE197945	Serum deprivation response	*Sdpr*	8.51	May play a role in regulation of **cell proliferation** and *Sdpr* induction appears to be coupled to **growth arrest**. SDPR is a substrate for protein kinase C (PKC) phosphorylation.
AK004781	SRY-box containing gene 17	*Sox17*	8.15	Sox17 is a transcription factor that regulates **cell cycle** and **proliferation**.
BB039247	CD93 antigen	*Cd93*	8.13	CD93 antigen plays a role in intercellular adhesion and in clearance of **apoptotic cells**.
BG917242	Lipoma HMGIC fusion partner-like 2	*Lhfpl2*	8.10	A member of the lipoma HMGIC fusion partner (LHFP) gene family, which is a subset of the superfamily of tetraspan transmembrane protein encoding genes.
NM_133654	CD34 antigen	*Cd34*	7.90	Possible adhesion molecule with a role in early hematopoiesis by mediating the attachment of stem cells to the bone marrow extracellular matrix or directly to stromal cells. Could act as a scaffold for the attachment of lineage specific glycans, allowing stem cells to bind to lectins expressed by stromal cells or other marrow components. Presents carbohydrate ligands to selectins.
BF100813	Endothelin receptor type B	*Ednrb*	7.10	Non-specific receptor for endothelin 1, 2, and 3. *Ednrb-/-* mice exhibit neurodegeneration suggesting *Ednrb* is important for **cell survival**; however this did not involve canonical **apoptosis signals**.
NM_011582	Thrombospondin 4	*Thbs4*	7.01	Adhesive glycoprotein that mediates cell-to-cell and cell-to-matrix interactions. C-terminal peptide can stimulate **cell proliferation.**
NM_133736	Regulator of G-protein signaling 5	*Rgs5*	6.92	Inhibits signal transduction and acts as a hypoxia-inducible **apoptotic** stimulator. Plays a role in mediating responses to **oxidative stress** through the MEK-ERK1/2 signalling pathway.
AW543698	Cadherin 5	*Cdh5*	6.70	A calcium-dependent cell–cell adhesion glycoprotein that plays an important role in **cell polarity** through control of the cohesion and organization of the intercellular junctions. It associates with alpha-catenin forming a link to the cytoskeleton. **Oxidative stress** can modulate Wnt/b-catenin and CDH5 in diabetes.
BC002076	Mesenchyme homeobox 2	*Meox2*	6.69	MEOX2 (mesodermal homeobox-2) also known as GAX (Growth Arrest-specific homeoboX) encodes a homeodomain-containing transcription factor that regulates NF-κB activity and induces cell cycle arrest. Interaction between NF-κB complex and MEOX2 through its homeodomain and N-terminal

				domain.
BC020152	Integrin, beta-like 1	*Itgbl1*	6.62	Proposed to participate in cell adhesion, cell-matrix interactions and integrin-mediated signalling pathways. Along with *Gpha2* it is up regulated by dexamethasone of murine cochlear explants
NM_019397	EGF-like-domain, multiple 6	*Egfl6*	6.55	Mediates a paracrine mechanism of cross-talk between vascular endothelial cells and osteoblasts, promoting **cell migration** and angiogenesis during bone development, grow and repair.

Supp. Table S4. The top 20 most significantly down regulated genes in chondrocytes from new born *Comp* p.D469del$^{m/m}$ mutant mice relative to wild type

Gene ID	Gene Name	Gene Symbol	Fold change	Putative role in PSACH pathology
NM_010743	Interleukin 1 receptor-like 1	*Il1rl1*	-6.09	Receptor for interleukin-33 (IL-33). Possibly involved in helper T-cell function.
NM_013653	Chemokine (C-C motif) ligand 5	*Ccl5*	-4.56	Chemoattractant for blood monocytes and induces **cell proliferation**. Involved in the **NF-kappa-B** signalling cascade.
AV347903	Guanine nucleotide binding protein (G protein), gamma 4 subunit	*Gng4*	-3.91	Involved as a modulator or transducer in various transmembrane **signalling** systems.
AF065917	Leukemia inhibitory factor	*Lif*	-3.90	Cytokine of the interleukin 6 superfamily that can activate the gp130-JAK-STAT3 signalling cascade and WNT5a signalling that enhances **cell proliferation**.
AK011963	Peroxiredoxin 2	*Prdx2*	-3.89	An anti-oxidant enzyme involved in redox regulation of the cell and reduces peroxides through the thioredoxin system. Peroxiredoxin 2 can inhibit cell **apoptosis** through the **NF-kappa-B** pathway.
BE852181	Rho family GTPase 1	*Rnd1*	-3.60	Multifunctional regulator of the cytoskeleton and **cell cycle progression**.
BB277065	Traf2 binding protein	*T2bp*	-3.46	Adapter protein which mediates IRAK1 and TRAF2 interaction, resulting in the downstream activation of **NF-kappa-B** and AP-1 pathways. Can play a role in **apoptosis** regulation. NfκB and Bcl2 pathway is blocked by induction of Tnf receptor-associated factor 2-inhibiting protein (Traip)
NM_010809	Matrix metallopeptidase 3	*Mmp3*	-3.35	*Mmp3*can degrade fibronectin, laminin, gelatins of type I, III, IV, and V; collagens III, IV, X, and IX, and cartilage proteoglycans.
BC015293	Gremlin 1	*Grem1*	-3.17	A cytokine that may play an important role during carcinogenesis and metanephric kidney organogenesis, as a BMP antagonist required for early limb outgrowth and patterning in maintaining the FGF4-SHH feedback loop. Down-regulates the BMP4 signalling in a dose-dependent manner.
NM_133232	6-phosphofructo-2-kinase/fructose-2,6-biphosphatase 3	*Pfkfb3*	-3.10	Protein involved in synthesis and degradation of fructose 2,6-bisphosphate. Can increase cell **proliferation** via cyclin-dependent kinases.
NM_054088	Patatin-like phospholipase domain containing 3	*Pnpla3*	-3.09	Multifunctional enzyme which has both triacylglycerol lipase and acylglycerol O-acyltransferase activities. Plays a role in **glucose metabolism**.
NM_007539	Bradykinin receptor, beta 1	*Bdkrb1*	-2.99	A receptor for bradykinin that can have a **protective role** in ischemia/riperfusion injury by reducing **apoptosis**.
NM_009140	Chemokine (C-X-C motif) ligand 2	*Cxcl2*	-2.94	CXCL2 mRNA induction can be mediated by p38, JNK and **NF-κB signalling** pathways. The knockdown of CXCL2 inhibits **proliferation** and ERK activation in bone marrow-derived macrophages.
BC019961	Chemokine (C-X-C motif) ligand 16	*Cxcl16*	-2.92	A pro-inflammatory cytokine, CXCL16/CXCR6 may play important roles in cell growth and proliferation Possibly involved in the translocation of nuclear factor-κB (**NF-κB**) p65 to the nucleus.
BB164127	Human immunodeficiency virus type I enhancer binding protein 3	*Hivep3*	-2.88	A zinc finger transcription factor, HIVEP3/KRC enhances **NF-κB** -dependent transactivation and JNK phosphorylation and therefore apoptosis and cytokine gene expression. The effect of HIVEP3/KRC is mediated through its interaction with the adaptor protein TRAF2, which intersects both pathways.
NM_009311	Tachykinin 1	*Tac1*	-2.81	*Tac1* encodes peptides belong to the tachykinin family of neuropeptides. **NF-κB** is implicated in the repression of *Tac1* at high levels of the CXC chemokine, stromal cell-derived factor-1alpha (SDF-1alpha).
BB185854	Alpha-2-macroglobulin	*A2m*	-2.75	Is able to inhibit all four classes of proteinases by a unique 'trapping' mechanism. Its MMPs inhibitory effect might play a role in skeletal development and homeostasis. *A2m* can induce JAK/STAT pathway activation.
AY078071	otospiralin	*Otos*	-2.73	Up regulation of *Otos* significantly **decreased apoptosis** through activation of ERK and partial inhibition of JNK and mitochondrial pathways.
BM210600	Sulfiredoxin 1 homolog (S. cerevisiae)	*Srxn1*	-2.72	Important in **oxidative stress** resistance by reducing cysteine-sulfinic acid formed under exposure to oxidants in the peroxiredoxins PRDX1, PRDX2, PRDX3 and PRDX4. *Srxn1* is a **NF-κB** regulated gene under both stressed and unstressed conditions.
BG072958	R-spondin 3 homolog (Xenopus laevis)	*Rspo3*	-2.69	The R-spondins (RSPOs) are a recently described family of secreted proteins that activate WNT and β-catenin signalling cascades. *Rspo3* binds syndecan 4 (Sdc4) and that together they activate Wnt and PCP signalling. Mice lacking *Rspo3* develop craniofacial abnormalities and have increased **cell apoptosis**.

Supp. Table S5. The top 20 most significantly up regulated genes in chondrocytes from 5 day old *Comp* p.D469del$^{m/m}$ mutant mice relative to wild type

Gene ID	Gene Name	Gene Symbol	Fold change	Putative role in PSACH pathology
AW910504	Ectonucleotide pyrophosphatase/ phosphodiesterase 1	*Enpp1*	4.11	Functions in bone mineralization and regulated by FGF2 in an ERK MAPK-dependent manner. A negative regulator of insulin receptor (IR) activation. Mutations cause hypophosphatemic rickets.
BB181225	Potassium voltage-gated channel, subfamily Q, member 5	*Kcnq5*	3.56	Role in potasium channel regulation which might be involved in **protection against apoptosis** induced by **oxidative stress** and also **cell proliferation**.
AV229143	Interferon activated gene 202B	*Ifi202b*	3.47	Interferon-inducible gene that along with survivin plays a role in **protection against apoptosis** and cell **proliferation**.
AI840829	Calcium/calmodulin-dependent protein kinase IV	*Camk4*	3.33	Calcium/calmodulin-dependent protein kinase that rgulates calcium-dependant gene expression and along with growth factor erv1-like (Gfer) **regulates cell proliferation**. Regulated by Wnt signalling and believed to have a cell protective role in Alzheimers and transient global ischemia.
BG072739	SRY-box containing gene 11	*Sox11*	3.16	Important for **cell survival** and growth and chondrogenesis.
AI324124	Synuclein, alpha	*Snca*	3.06	Reduces cells response to various **apoptotic stimuli** leading to decreased caspase-3 activation.
NM_008537	Alpha-methylacyl-CoA racemase	*Amacr*	2.73	Enzyme responsible for the racemization of branched-chain lipids. Important in **oxidative degradation pathways** and regulates entry into the peroxisomal and mitochondrial beta-oxidation pathways.
NM_010442	Heme oxygenase (decycling) 1	*Hmox1*	2.58	Has **anti-oxidative activities** and can be up regulated by hydrogen peroxide. May protect against anoxia/reoxygenation-induced **apoptosis**.
NM_007606	Carbonic anhydrase 3	*Car3*	2.50	Cytosolic zinc-containing metalloenzymes that catalyses reversible hydration of carbon dioxide and influence mineralization. Car3 can also respond to **oxidative stress** and has a protective role against hydrogen peroxide induced **apoptosis**.
BB084936	Arylsulfatase J	*Arsj*	2.46	Involved in hormone biosynthesis, modulation of cell signaling, and **degradation** of macromolecules.
AF260740	Glycoprotein hormone alpha 2	*Gpha2*	2.36	Stimulates the thyroid. Binds and activates THSR and increases cAMP production.
AV124537	Lymphatic vessel endothelial hyaluronan receptor 1	*Lyve1*	2.33	Plays a role in autocrine regulation of cell growth and may also act as a hyaluronan transporter. LYVE1 and thrombospondin-2 (THBS2) were identified as **mediators of IGFBP-3-dependent effects**.
BB035005	Multimerin 1	*Mmrn1*	2.30	Large, homopolymeric adhesive protein that plays a role in the storage and stabilization of factor V in platelets. May function as an extracellular matrix or adhesive protein. Neighbouring gene to **Snca** in the mouse genome
BG073776	Interleukin 1 receptor-like 2	*Il1rl2*	2.21	Receptor for interleukin 1 family member 9 (IL1F9). Binding to the agonist leads to the activation of **NF-κB**. May play a role in the pathogenesis of distal interphalangeal oasteoarthritis.
AV321547	Decorin	*Dcn*	2.21	Plays a role in matrix assembly. A **cell-cycle regulator** that can reduce cell **proliferation** by induction of **apoptosis**.
NM_009982	Cathepsin C	*Ctsc*	2.17	Thiol protease with dipeptidylpeptidase activity. Activate serine proteases making them capable of **degrading** various extracellular matrix components.
NM_010417	Hephaestin	*Heph*	2.16	Functions as a multicopper ferroxidase and over-expression can attenuate iron-induced **oxidative stress** *in vitro*.
U73910	Phosphate regulating gene with homologies to endopeptidases on the X chromosome	*Phex*	2.16	A zinc endopeptidase involved in bone mineralization and mutations cause hypophosphatemic rickets. **NF-κB** signalling and PARP-1 enzymatic activity cooperatively contributes to the constitutive suppression of Phex.
NM_007727	Contactin 1	*Cntn1*	2.08	Contactins are membrane anchored cell adhesion molecules that mediate **cell surface interactions** during development via interaction with chondroitin sulfate E.
NM_007429	Angiotensin II receptor, type 2	*Agtr2*	2.07	Receptor for angiotensin II and cooperates with MTUS1 to inhibit ERK2 activation and **cell proliferation**. *Agtr2* induces **NF-κB**-mediated transcription and expression of **NF-κB** -related genes.

Supp. Table S6. The top 20 most significantly down regulated genes in chondrocytes from 5 day old *Comp* p.D469del$^{m/m}$ compared to wild type

Gene ID	Gene Name	Gene Symbol	Fold change	Putative role in PSACH pathology
AA210261	DEAD (Asp-Glu-Ala-Asp) box polypeptide 3, Y-linked	*Ddx3y*	-7.93	DEAD-box RNA helicases have been implicated a variety of processes that regulate gene expression, such as transcription, splicing, mRNA export and translation. It is also proposed to be involved in **cell cycle** control and the regulation of **apoptosis**.
AV348246	Neuronal PAS domain protein 4	*Npas4*	-4.48	Transcription factor that is implicated in adaptation to cellular and **oxidative stresses**.
BB656631	SRY-box containing gene 11	*Sox11*	-3.53	Probably important in the developing nervous system. May also have a role in lymphocyte proliferation.
NM_012011	Eukaryotic translation initiation factor 2, subunit 3, structural gene Y-linked	*Eif2s3y*	-3.15	Along with *Ddx3y* it is induced following angiotensin II treatment of mice. *Eif2s3y* functions in the early steps of protein synthesis by forming a ternary complex with GTP and initiator tRNA.
AK011963	Peroxiredoxin 2	*Prdx2*	-2.91	An anti-oxidant enzyme involved in redox regulation of the cell and reduces peroxides through the thioredoxin system. Peroxiredoxin 2 can inhibit cell **apoptosis** through the **NF-kappa-B** pathway.
BB218576	calcium/calmodulin-dependent protein kinase II, delta	*Camk2d*	-2.74	Angiotensin II-induced oxidative stress resets the Ca2+ dependence of *Camk2d* and promotes cell death. Inhibition of *Camk2d* prevents an increase in p53 and **apoptosis**.
NM_133743	Ly6/Plaur domain containing 3	*Lypd3*	-2.67	Supports **cell migration** and may be involved in cell-matrix **interactions**.
NM_008127	Gap junction protein, beta 4	*Gjb4*	-2.44	Gap junction protein that is also known as connexin Cx30.3 and though to mediate cell-cell communication.
AU046270	Acireductone dioxygenase 1	*Adi1*	-2.38	***Adi1*** is a member of the Cupin superfamily, which binds to and inhibits the activities of membrane-type 1 matrix metalloproteinase (MMP14). Increased *Adi1* expression can cause **apoptosis**.
BC004633	NADH dehydrogenase (ubiquinone) 1 alpha subcomplex, 12	*Ndufa12*	-2.20	Accessory subunit of the **mitochondrial** membrane respiratory chain NADH dehydrogenase (Complex I)
AV128350	Block of proliferation 1	*Bop1*	-2.14	Component of the Pes1-Bop1-WDR12 (PeBoW) complex, which is required for maturation of 28S and 5.8S ribosomal RNAs and formation of the 60S ribosome. May play a role in cytoskeletal remodelling. PeBoW complex is required for ribosome biogenesis and **cell proliferation**.
BF148215	Strawberry notch homolog 2 (Drosophila)	*Sbno2*	-2.12	*Sbno2* has transcriptional repression activity and has been shown to repress **NF-kappa-B** signalling.
NM_009864	Cadherin 1	*Cdh1*	-2.11	Involved in mechanisms regulating cell-cell adhesions, mobility and **proliferation** of epithelial cells. Has a potent invasive suppressor role and is a ligand for integrin alpha-E/beta-7.
L07264	Heparin-binding EGF-like growth factor	*Hbegf*	-2.09	**Cell migration** can be mediated by MMP7-mediated release of HBEGF that activates ERBB1 and downstream ERK and PI3K **signalling**.
NM_053015	Melanophilin	*Mlph*	-2.07	Rab effector protein involved in melanosome transport. The actin-activated MgATPase activity of myosin 5a is activated by binding to melanophilin. Myosin 5 is a motor protein that moves cargoes along actin filaments.
BB486599	ST8 alpha-N-acetyl-neuraminide alpha-2,8-sialyltransferase 6	*St8sia6*	-2.06	Belongs to a family of sialyltransferases that synthesize sialylglycoconjugates. Prefers O-glycans to N-glycans or glycolipids as acceptor substrates.
AK009828	Neuraminidase 2	*Neu2*	-2.06	Remove sialic acid residues from glycoproteins and glycolipids.
AK019319	Apolipoprotein E	*Apoe*	-2.06	Mediates the binding, internalization, and catabolism of lipoprotein particles.
BF322712	3-hydroxybutyrate dehydrogenase, type 1	*Bdh1*	-2.04	Catalyzes the interconversion of acetoacetate and (R)-3-hydroxybutyrate, the two major ketone bodies produced during fatty acid catabolism.
AW553130	Triple functional domain (PTPRF interacting)	*Trio*	-2.04	Promotes the exchange of GDP by GTP. Together with leukocyte antigen-related (LAR) protein, it could play a role in coordinating cell-matrix and cytoskeletal rearrangements necessary for **cell migration** and cell growth.
